# Composition and natural history notes of the coastal snake assemblage from Northern Bahia, Brazil

**DOI:** 10.3897/zookeys.611.9529

**Published:** 2016-08-15

**Authors:** Ricardo Marques, Konrad Mebert, Érica Fonseca, Dennis Rödder, Mirco Solé, Moacir Santos Tinôco

**Affiliations:** 1 Universidade Federal da Paraíba, Departamento de Sistemática e Ecologia, Programa de Pós-Graduação em Ciências Biológicas (Zoologia). Cidade Universitária. Rua José Dionísio da Silva, s/n, 58059-900, João Pessoa, PB, Brazil; 2 Universidade Estadual de Santa Cruz, Programa de Pós-Graduação em Zoologia. Rodovia Jorge Amado, km 16. CEP 45662-900. Ilhéus, BA, Brazil; 3 Universidade Federal de Santa Maria, Programa de Pós-Graduação em Biodiversidade Animal. Avenida Roraima, n° 1000, Cidade Universitária. CEP 67105-900. Camobi, Santa Maria, RS, Brazil; 4 Zoological Research Museum Alexander Koenig, Department of Herpetology, Adenauerallee 160, 53113 Bonn, Germany; 5 University of Kent at Canterbury; DICE - Durrell Institute of Conservation and Ecology; School of Anthropology and Conservation. Marlowe Building, Kent, CT2 7NZ, UK; 6 Universidade Católica do Salvador, PROPP-PPGPA - Centro de Ecologia e Conservação Ambiental – ECOA. Avenida Prof. Pinto de Aguiar, 2589. CEP 41740-090. Pituaçu, Salvador, BA, Brazil

**Keywords:** Atlantic forest, coastal sand dunes, conservation, ombrophilous forest, restinga, snakes, conservation

## Abstract

Information about the snake diversity and their natural history from the Atlantic forest domain in Brazil refer mostly to inland forests than to coastal region. Within the state of Bahia, this knowledge is concentrated to the southeastern coastal stretch. Herein we report on the diversity of snakes from the restinga, ombrophilous forest and anthropogenic environment from the northern Atlantic coast of Bahia. We sampled nine sites for three years and visited four museum collections. Furthermore, we provide anecdotal natural history information, voucher analyses, literature complements, and a key to fascilitate species identification. We report a total of 774 snakes belonging to 50 species and 23 new distribution records for northeastern coast of Bahia, supplemented by new data on feeding and reproduction. The number of detected species is similar to numbers obtained in comparable studies from other Brazilian ecoregions. This study reports and focuses for the first time on all known species of snakes from the northeastern coast of Bahia.

## Introduction

Studies on diversity inventories and natural history increase the knowledge of a regional fauna, its interaction with other organisms, and the environment in general (Greene 1994). As such, they provide relevant basis data to better understand the complexity of ecosystems. This is not different with snakes, which represent an integral part of such ecosystems, be them as prey, predator, host and other functions within the network of ecosystem relations. Therefore, snakes, as well as other constituents of a local ecosytems, are important and need to be understood and protected. Hence, novel data about our target group is elementary for further conservation studies on these taxa (Greene and Losos 1988, Shine and Bonnet 2009).

As a first step, pertinent and relevant taxa-related information can be acquired through species inventories and studies describing aspects of natural history that increase our knowledge on the different habitats and habits of regional snakes (Greene 1997). These studies include biodiversity components in terms of richness, abundance, distribution, diet composition, activity periods, reproduction, morphological variation, parasitism, predators and other intrinsic data of the group (Martins and Oliveira 1998). All these aspects enhance the knowledge of snakes (Cadle and Greene 1993), but these studies are not evenly distributed among the different Brazilian ecoregions. For example, there are several contributions on snake communities from the Amazon (e.g. Cunha and Nascimento 1978, Martins and Oliveira 1998, Santos-Costa et al. 2015), as well as the Cerrado (e.g. Recoder and Nogueira 2007, Sawaya et al. 2008), Caatinga (e.g. Vanzolini et al. 1980, Vitt and Vanglinder 1983, Guedes et al. 2014) and Pantanal (e.g. Strüssmann and Sazima 1993). Regarding the Atlantic forest, the snake fauna from the Southeast and South of Brazil is well represented (e.g. Marques et al. 2001, Marques and Sazima 2004, Hartmann et al. 2009a, b), but despite the increase of these studies in recent years, the natural history of these animals from most northeastern states of Brazil has remained poorly documented.

Studies involving snakes from northeastern Brazil are often species inventories from the Caatinga and transition areas with other ecoregions (Vanzolini et al. 1980, Vitt and Vangilder 1983, Pereira Filho and Montingelli 2011, Loebmann and Haddad 2010, Ribeiro et al. 2012, Garda et al. 2013, Mesquita et al. 2013, Guedes et al. 2015). Unfortunately, the Atlantic forest in northeastern Brazil is represented by few studies only (Argôlo 2004, Santana et al. 2008, Morato et al. 2011, França et al. 2012), rendering a regional study, such as the one presented herein, the more important. A total of 131 snake species are currently known from the state of Bahia (Hamdan and Lira-da-Silva 2012, Curcio et al. 2012, Fernandes and Hamdan 2014), but there is a lack of detailed studies. Argôlo (2004) provides the most complete compilation of the snake assemblage of the Atlantic forest in southern Bahia, reporting species from different forested, disturbed and open habitats. Dias and Rocha (2014) surveyed the herpetofauna from restinga in the northern and southern coast of the state, while Marques et al. (2011) recorded snakes from a single restinga location in the northern coast of Bahia. Herein we include: (1) a checklist of all snake species from the northern coast of Bahia; (2) natural history information (frequency, size, distribution, habitat, microhabitat, activity and environmental variables, diet, reproduction and defensive behavior); (3) conservation aspects, and (4) an identification key to the snakes from this region.

## Material and methods

### Study area

**Figure 1.**
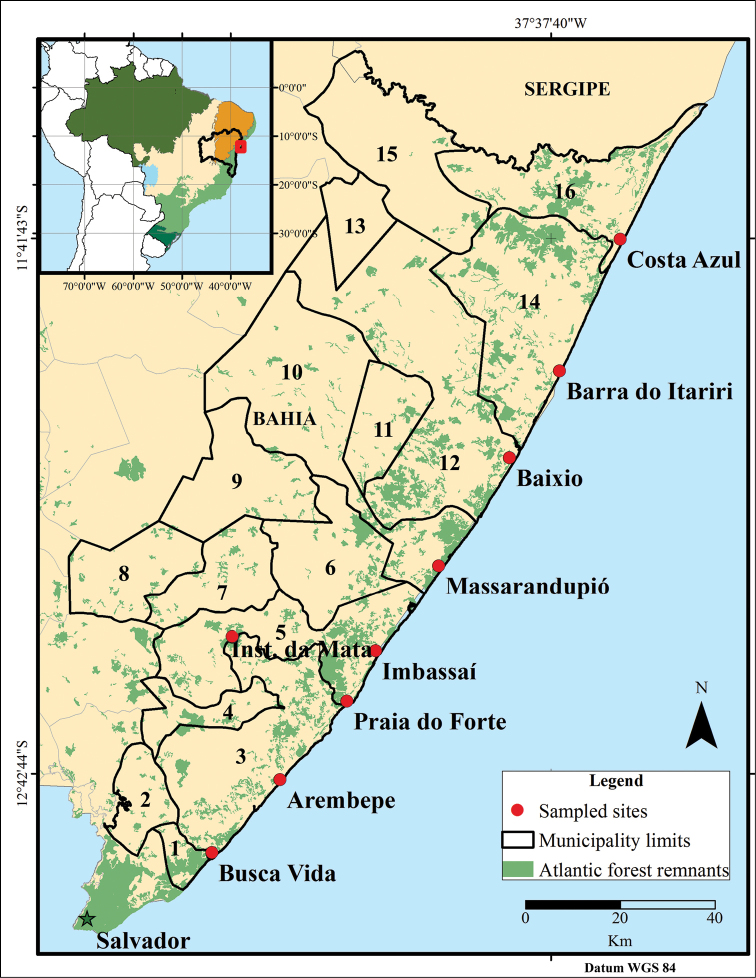
The northern coast of Bahia and municipalities of the study: **1** Lauro de Freitas **2** Simões Filho **3** Camaçari **4** Dias D’Ávila **5** Mata de São João **6** Itanagra **7** Pojuca **8** Catu **9** Araçás **10** Entre Rios **11** Cardeal da Silva **12** Esplanada **13** Acajutiba **14** Conde **15** Rio Real **16** Jandaíra.

**Figure 2.**
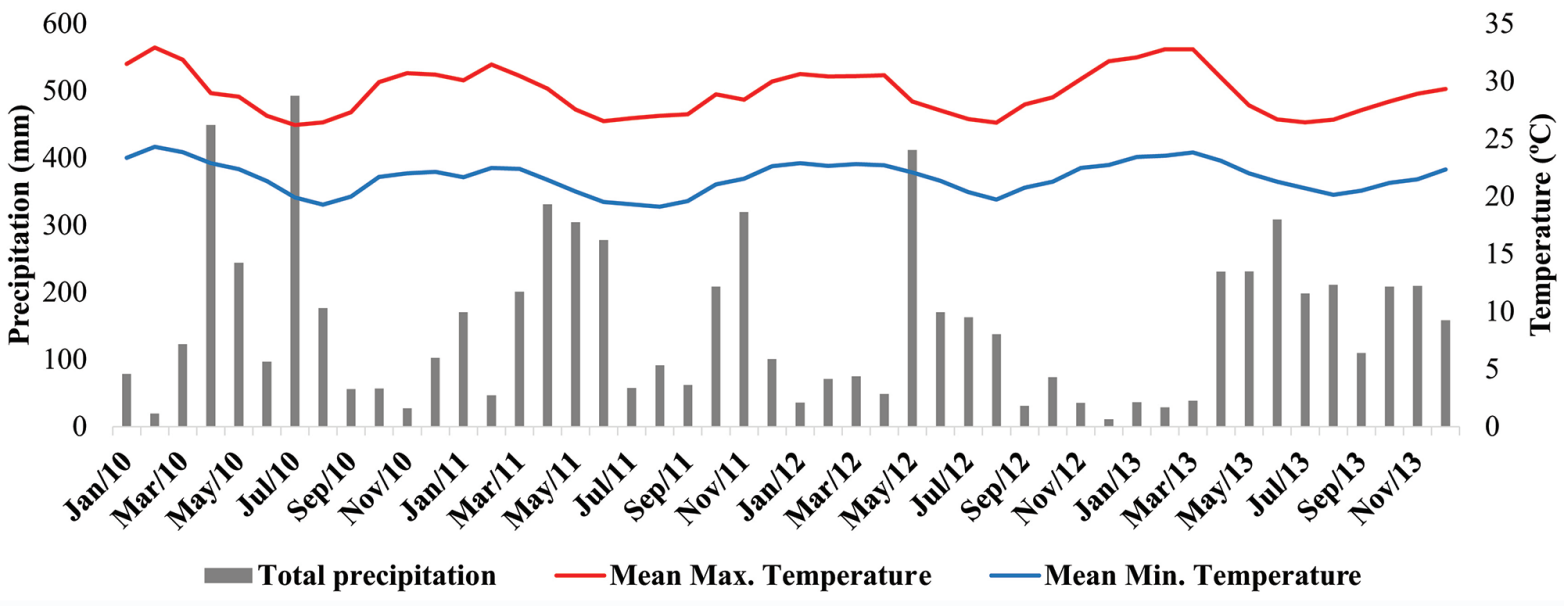
Variation of temperature, maximum (red) and minimum (blue), precipitation as bars. Data from January 2010 and December 2013 (Source: INMET).

The present study was conducted on the northern coast of Bahia, which extends for 220 km from Salvador to the boundaries of the state of Sergipe (Figure 1). The climate in this region varies from humid to subhumid with a weak dry season in the southernmost portion and a two months dry season in the remaining area. Precipitation fluctuates from 1300 to 1900 mm per year (Figure 2) (IBGE 2002, SEI 2013). The vegetation in the coastal municipalities (next lower subdivision of the state) consists of pioneer vegetation with marine influence, called restinga. Four typical vegetation types compose the restinga of the region: beach vegetation, flooded plain, shrub vegetation and restinga dry forest (Menezes et al. 2009, 2012). Farther inland, the landscape changes to dense ombrophilous forests and semideciduous stationary forests, both with Cerrado enclaves (SEI 2013).

### Sample design

**a) Fieldwork**

Snakes were sampled during 18 bimonthly fieldtrips from 2010 to 2013, covering three dry seasons and three rainy seasons, totaling in 162 field days. We sampled eight coastal sites of the restinga ecosystem: Busca Vida (-12.8619 S, -38.2708 W), Arembepe (-12.7236 S, -38.1416 W), Praia do Forte (-12.5748 S, -38.0147 W), Imbassaí (-12.4791 S, -37.9602 W), Massarandupió (-12.3172 S, -37.8404 W), Baixio (-12.1123 S, -37.7062 W), Barra do Itariri (-11.9478 S, -37.6113 W) and Costa Azul (-12 S, -37.496 W); and the Instituto da Mata, an ombrophilous forest fragment of 410 ha at 20 km from coast (-12.450073 S, -38.234579 W). Surveys were authorized under ICMBio No 23355-2.

Four sampling sources were used to acquire data on snakes: (1) Visual Encounter Survey (VES) limited by time, i.e. recording visually detected and manually captured snakes; (2) dead specimens found by locals; (3) carcasses and shed skins detected by us; and (4) incidentally encountered and recorded snakes outside the VES. For the VES, we walked for two hours on four 500 m transects placed on four aforementioned vegetation types. The VES covered the period from 06:00 h and 18:00 h in six turns: 06:00–08:00; 08:00–10:00; 10:00–12:00; 12:00–14:00; 14:00–16:00; 16:00–18:00. In the last year VES was performed during nighttime from 19:00 h to 21:00 h at Busca Vida, Baixio, Barra do Itariri and Instituto da Mata, totaling a sample effort of 3024 h (2592 h diurnal and 432 h nocturnal). During the VES and incidental encounters we recorded for each snake the time of activity, predominant vegetation type and microhabitat. Each captured snake was marked and then released at its site of capture. Marking involved fluorescent elastomer applied subcutaneously in the ventral scales. The i^th^ number of the ventral scale, with the first number being the ventral scale anterior the anal plate, corresponding to the i^th^ number of the captured snake (adapted from Hutchens et al. 2008). We used four different colors, one for each vegetation type to see if animals were moving among the vegetation types in case of recapture. Voucher specimens are deposited in the Coleção Herpetológica do Centro de Ecologia e Conservação Animal at the Universidade Católica do Salvador.

**b) Animal characterization**

All species were identified according to the literature (Peters and Orejas-Miranda 1970; Vanzolini et al. 1980, Cunha and Nascimento 1978, Dixon 1986, 1989, Dixon et al. 1993, Campbell and Lamar 2004, Fernandes 2006, Albuquerque 2008, Lima 2012, Ascenso 2013, Pires et al. 2014).


 Snout-Vent Length (SVL) and sex was recorded from all captured animals and museum specimens when possible. We observed predation events, and during the last year of surveys, we forced animals to regurgitate their stomach content through manual palpation.

Morphological characteristics of the animals from the region comprising scale counts, body shape and coloration allowed us to elaborate an identification key to all species recorded in this study. We complemented the information of animals with records obtained from the literature, as well as field observations and data from voucher specimens. We did not include natural history notes of introduced (non-native) species.

**c) Data collection in museums**

Specimens of four scientific collections from the state of Bahia were analysed: Museu de Zoologia at Universidade Estadual de Santa Cruz
(MZUESC, Ilhéus); Coleção herpetológica do Museu de Zoologia at Universidade Federal da Bahia (MZUFBA, Salvador); Coleção Herpetológica do Museu de Zoologia at Universidade Estadual de Feira de Santana
(MZUEFS, Feira de Santana); and Coleção Herpetológica de Referência do Centro de Ecologia e Conservação Ambiental at Universidade Católica do Salvador (CHECOA, Salvador). Considering the extensive region and lack of surveys from northeastern Bahia, and in particular its inland portions, we included additional records using voucher specimens from 10 neighbouring municipalities: Lauro de Freitas (-12.869 S, -38.315 W), Simões Filho (-12.768 S, -38.407 W), Dias D’Ávila (-12.5888 S, -38.269 W), Pojuca (-12.410 S, -38.321 W), Catu (-12.323 S, -38.420 W), Itanagra (-12.302 S, -38.059 W), Araçás (-12.16 S, -38.123 W), Cardeal da Silva (-12.072 S, -38.98 W), Acajutiba (-11.670 S, -38.017 W) and Rio Real (-11.502 S, -37.955 W) (Figure 1). No snakes were obtained from the municipalities of Araçás, Acajutiba, Cardeal da Silva and Rio Real. When possible, stomach contents and reproductive status were verified. Vouchers are listed in Appendix 1.

### Statistics

Species richness and frequency were computed based on the species sampled during fieldwork only, excluding specimens housed in collections. The frequency of the species from the northern coast of Bahia followed the model adapted by Mesquita et al. (2013) (extracted from Abreu and Nogueira 1989 and Luiselli 2006). The frequency of a certain snake species is computed by dividing the number of samples (18 bimonthly field trips) containing the target species by the total number of samples × 100. The resulting percentage classified the species as accidental (0.1%-25%), accessory (25%-50%) or constant (50%-100%). We calculated the dominance with the number of specimens of the target species divided by the total number of snakes recorded during fieldwork × 100, applying the categories: accidental (0%-2.5%), accessory (2.5%-5%) and dominant (5%-100%). The combination of frequency and dominance enables the classification of the species as: 1) very rare (accidental/accidental, dominance < 1%), 2) rare (accidental/accidental), 3) intermediate (constant/accessory, constant/accidental, accessory/accidental, accessory/dominant, accessory/accessory), and 4) common (constant/dominant).

## Results

A total of 194 snakes were recorded during fieldwork and 580 obtained from museum collections, totaling 774 snakes of a total of 49 native species. Of those, 32 species were recorded during fieldwork and 17 species were complemented through museum records and literature references. Twenty-three new distributional records of species are added for the northern coastal stretch of Bahia (see Table 1 for details). In the municipality of Camaçari one specimen of an introduced species, *Pantherophis guttatus*, which is native to North America (see Fonseca et al. 2014) was detected. As it is non-native, any natural history descriptions were excluded, but included in the frequency statistics.

**Table 1.** Snake species from the northern coast of Bahia. Number of specimens from fieldwork (N), frequency (f%), dominance (D%) and number of specimens from museum specimens (NC). New records (*); Introduced species (**); Extracted from Dias and Rocha (2014) (***).

Species	N	f%	D%	NC
**Infraorder Scolecophidia Cope, 1864**	-	-	-	-
**Typhlopidae Merrem, 1820**	-	-	-	-
*Amerotyphlops brongersmianus* (Vanzolini, 1976)*	1	5.55	0.51	6
**Infraorder Henophidia Nopcsa, 1923**				
**Boidae Gray, 1825**				
**Boinae Gray, 1825**				
*Boa constrictor constrictor* Linnaeus, 1758	19	72.22	9.79	4
*Corallus hortulanus* (Linnaeus, 1758)	1	5.55	0.51	-
*Epicrates assisi* Machado, 1945*	-	-	-	1
*Eunectes murinus* (Linnaeus, 1758)	3	16.67	1.54	2
**Caenophidia Hoffstetter, 1939**				
**Colubridae Oppel, 1811**				
*Chironius bicarinatus* (Wied, 1820)***	-	-	-	-
*Chironius carinatus* (Linnaeus, 1758)*	2	11.1	1.03	-
*Chironius exoletus* (Linnaeus, 1758)	1	5.55	0.51	13
*Chironius flavolineatus* (Jan, 1863)	22	72.22	11.34	16
*Drymarchon corais corais* (Boie, 1827)*	-	-	0.51	2
*Drymoluber dichrous* (Peters, 1863)*	-	-	0.51	4
*Leptophis ahaetulla liocercus* (Wied, 1824)*	1	5.55	0.51	3
*Mastigodryas bifossatus* (Raddi, 1820)*	-	-	-	4
*Oxybelis aeneus* (Wagler, 1824)	6	22.22	3.09	5
*Pantherophis guttatus* (Linnaeus, 1766)**	-	-	-	1
*Spilotes pullatus pullatus* (Linnaeus, 1758)	2	11.11	1.03	7
*Spilotes sulphureus sulphureus* (Wagler, 1824)	1	5.55	0.51	-
*Tantilla melanocephala* (Linnaeus, 1758)*	2	11.11	1.03	9
**Dipsadidae Bonaparte, 1838**				
**Dipsadinae Bonaparte, 1838**				
**Dipsadini Bonaparte, 1838**				
*Sibynomorphus neuwiedi* (Ihering, 1911)	2	11.11	1.03	13
**Imantodini Myers, 2011**				
*Imantodes cenchoa cenchoa* (Linnaeus, 1758)	1	5.55	0.51	1
*Leptodeira annulata annulata* (Linnaeus, 1758)*	5	22.22	2.57	21
**Xenodontinae Bonaparte, 1845**				
**Echinantherini Zaher, Grazziotin, Cadle, Murphy, Moura-Leite & Bonatto, 2009**				
*Taeniophallus occipitalis* (Jan, 1863)	5	27.78	2.57	4
**Hydropsini Dowling, 1975**				
*Helicops angulatus* (Linnaeus, 1758)*	11	27.78	5.67	10
*Helicops leopardinus* (Schlegel, 1837)*	1	5.55	0.51	14
**Philodryadini Cope, 1886**				
*Philodryas nattereri* Steindachner, 1870	38	83.33	19.58	11
*Philodryas olfersii herbeus* Wied, 1825	9	33.33	4.63	13
*Philodryas patagoniensis* (Girard, 1858)	14	50	7.21	18
**Pseudoboini Bailey, 1967**				
*Clelia plumbea* (Wied, 1820)*	-	-	-	3
*Oxyrhopus petolarius digitalis* Reuss, 1834*	2	11.11	1.03	3
*Oxyrhopus trigeminus* Duméril, Bibron & Duméril, 1854	9	33.33	4.63	46
*Phimophis guerini* (Duméril, Bibron & Duméril, 1854)	-	-	-	4
*Pseudoboa nigra* (Duméril, Bibron & Duméril, 1854)*	-	-	-	7
*Siphlophis compressus* (Daudin, 1803)	1	5.55	0.51	-
**Thachymenini Bailey, 1967**				
*Thamnodynastes pallidus* (Linnaeus, 1758)	1	5.55	0.51	-
**Xenodontini Bonaparte, 1845**				
*Erythrolamprus aesculapii venustissimus* Wied, 1821*	-	-	-	1
*Erythrolamprus almadensis* (Wagler, 1824)*	2	11.11	1.03	17
*Erythrolamprus miliaris merremi* (Wied, 1821)*	-	-	-	3
*Erythrolamprus poecilogyrus schotti* (Schlegel, 1837)*	-	-	-	21
*Erythrolamprus reginae semilineatus* (Wagler, 1824)	1	5.55	0.51	8
*Erythrolamprus taeniogaster* (Jan, 1863)	1	5.55	0.51	12
*Erythrolamprus viridis viridis* (Günther, 1862)*	-	-	-	1
*Xenodon merremii* (Wagler, 1824)*	1	5.55	0.51	23
*Xenodon rabdocephalus rabdocephalus* (Wied, 1824)*	-	-	-	2
**Elapidae Boie, 1827**				
**Elapinae Boie, 1827**				
*Micrurus corallinus* (Merrem, 1820)*	-	-	-	1
*Micrurus ibiboboca* (Merrem, 1820)	16	55.56	8.24	81
**Viperidae Laurenti, 1768**				
**Crotalinae Oppel, 1811**				
*Bothrops erythromelas* Amaral, 1923	-	-	-	2
*Bothrops leucurus* Wagler, 1824	10	44.44	5.15	146
*Bothrops lutzi* (Miranda-Ribeiro, 1915)	-	-	-	3
*Crotalus durissus cascavella* Wagler, 1824	3	16.67	1.54	13
*Lachesis muta rhombeata* Wied-Neuwied, 1824*	-	-	-	1
**Total number of specimens**	194	-	100%	580

The most diverse family was Dipsadidae (25 spp.), representing 50% of the records. The second most diverse family was Colubridae (13 spp.), followed by Viperidae (5 spp.), Boidae (4 spp.), Elapidae (2 spp.) and Typhlopidae (1 sp.). Among the recorded species *Philodryas nattereri* (n = 38), *Chironius flavolineatus* (n = 22) and *Boa constrictor* (n = 19) were the most frequent species in restinga habitat, whereas *Helicops angulatus* (n = 11) dominated ombrophilous forest edges.

*Epicrates assisi*, *Drymarchon corais corais*, *Drymoluber dichrous*, *Mastigodryas bifossatus*, *Clelia plumbea*, *Phimophis guerini*,﻿ *Pseudoboa nigra*, *Erythrolamprus aesculapii venustissimus*, *Erythrolamprus miliaris merremii*, *Erythrolamprus poecilogyrus schotti*, *Erythrolamprus viridis viridis*, *Xenodon rhabdocephalus rhabdocephalus*, *Micrurus corallinus*, *Bothrops erythromelas*, *Bothrops lutzi* and *Lachesis muta rhombeata* were detected only through museum vouchers, and appear to be rare (n ≤ 4), except for *Erythrolamprus poecilogyrus schotti* (n = 21) and *Pseudoboa nigra* (n = 7).

**Figure 3.**
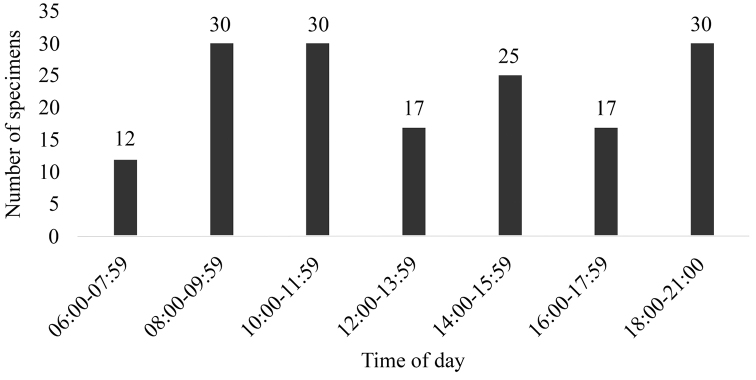
Activity of snakes recorded during the day.

Diurnal activity was recorded for 158 snakes during all surveys with members of the families Typhlopidae, Colubridae (except *Tantilla melanocephala*) and Elapidae restricted to diurnal activity. Our observations show snakes were mostly active during morning between 08:00 h and 11:59 h and less frequently observed during the afternoon (Figure 3). During the hottest period of the day (12:00 h – 14:00 h) we recorded 17 snakes; eight of those were *Philodryas nattereri* foraging on the ground. This species might tolerate high temperatures, since we observed an adult (SVL ≥ 800 mm) moving on the sandy soil at 55 °C. We present occasional encounters and nighttime surveys from the last year (18:00 h-21:00 h; Figure 3), when we observed mostly *Helicops angulatus*, *Leptodeira annulata*, *Oxyrhopus* spp. and viperids. We also recorded 13 snakes on roads of which four were killed by cars.

### Food items

Stomach contents of 11 species were analysed. Lizards represented 40% of food items, followed by amphibians (20%), mammals and snakes (15% each), fishes and invertebrates (5% each). The lizard *Ameivula ocellifera* was the most recurrent prey item in restinga with observed predatory events between 09:50 h and 13:06 h. Two isolated events of *Oxyrhopus trigeminus* feeding on *Ameivula ocellifera* were recorded, when the snakes (SVL = 320 mm and 315 mm) preyed on three lizards (SVL = 53.8 mm and 51.9 mm; a partially digested specimen, respectively). Another lizard species ingested by *Oxyrhopus trigeminus* was *Tropidurus hygomi*. In addition, *Ameivula ocellifera* was also a food item of *Philodryas nattereri* in two different events. In the first, the snake (SVL = 390 mm) regurgitated a partially digested *Ameivula ocellifera*. In the second event, an adult *Philodryas nattereri* subdued the lizard and disappeared immediately thereafter with it. We report the first predatory event of the alien species *Hemidactylus mabouia* (SVL = 41.5 mm) by *Chironius flavolineatus* (SVL = 610 mm). The viper *Bothrops leucurus* collected in a nearby anthropogenic construction site at the Instituto da Mata contained *Tropidurus hispidus* remnants in its stomach.

Amphibians were the second most frequent food item. An unidentified tadpole (total lenght = 39.4 mm) was ingested by a juvenile *Chironius exoletus* (SVL = 190 mm). We recorded the ingestion of *Leptodactylus natalensis* by *Helicops angulatus* in two events, both partially digested. One of these events also revealed frog eggs, possibly of the same prey species. Herein we report the first predation record of *Amerotyphlops brongersmianus* (SVL = 321.1 mm) by *Micrurus ibiboboca* (SVL = 558 mm) and the first cannibalism event of *Oxyrhopus trigeminus* in which a specimen (SVL = 390 mm) ingested a juvenile (SVL approximately 140 mm). We list all food items and new predatory records in Table 2.

**Table 2.** Stomach content of snake from the northern coast of Bahia, number of items found by species (N). New records for this species’ diet (*).

Species	N	Food item
**Colubridae**		
*Chironius flavolineatus*	1	*Hemidactylus mabouia* (Squamata, Gekkonidae)*
*Chironius exoletus*	1	Tadpole
**Dipsadidae**		
*Sibynomorphus neuwiedi*	1	Gastropod (Mollusca, Veronicellidae)
*Helicops angulatus*	2	*Leptodactylus natalensis* (Anura, Leptodactylidae)*
	1	Anuran eggs
*Helicops leopardinus*	1	*Geophagus brasiliensis* (Pisces, Cichlidae)
*Oxyrhopus trigeminus*	1	*Tropidurus hygomi* (Squamata, Tropiduridae)*
	3	*Ameivula ocellifera* (Squamata, Teiidae)
	1	*Oxyrhopus trigeminus* (Serpentes, Dipsadidae)*
*Philodryas nattereri*	2	*Ameivula ocellifera* (Squamata, Teiidae)
*Philodryas patagoniensis*	1	*Philodryas olfersii* (Serpentes, Dipsadidae)
**Elapidae**		
*Micrurus ibiboboca*	1	*Amerotyphlops brongersmianus* (Serpentes, Typhlopidae)*
**Viperidae**		
*Bothrops leucurus*	1	*Tropidurus hispidus* (Squamata, Tropiduridae)
	2	*Rattus norvegicus* (Mammalia, Muridae)
*Crotalus durissus*	1	*Rattus* sp. (Mammalia, Muridae)

### Natural history notes of species

#### TYPHLOPIDAE Merrem, 1820

***Amerotyphlops brongersmianus* Vanzolini, 1976**

Fig. 4A

A rare species, small sized (min SVL = 110 mm, max SVL = 315 mm). Recorded at Reserva Sapiranga, Imbassaí and Barra do Itariri. Inhabits ombrophilous forest and restinga dry forest, where a *Micrurus ibiboboca* predated a specimen on 06/03/2013. Marciano Junior et al. (2010) reported a bluish coloration in offsprings. This observation is confirmed from a young museum specimen (SVL ≤ 185 mm) with remnants of juvenile bluish coloration transitioning into brownish coloration. Strüssmann and Sazima (1993) reported insects as part of its diet.

**Figure 4.**
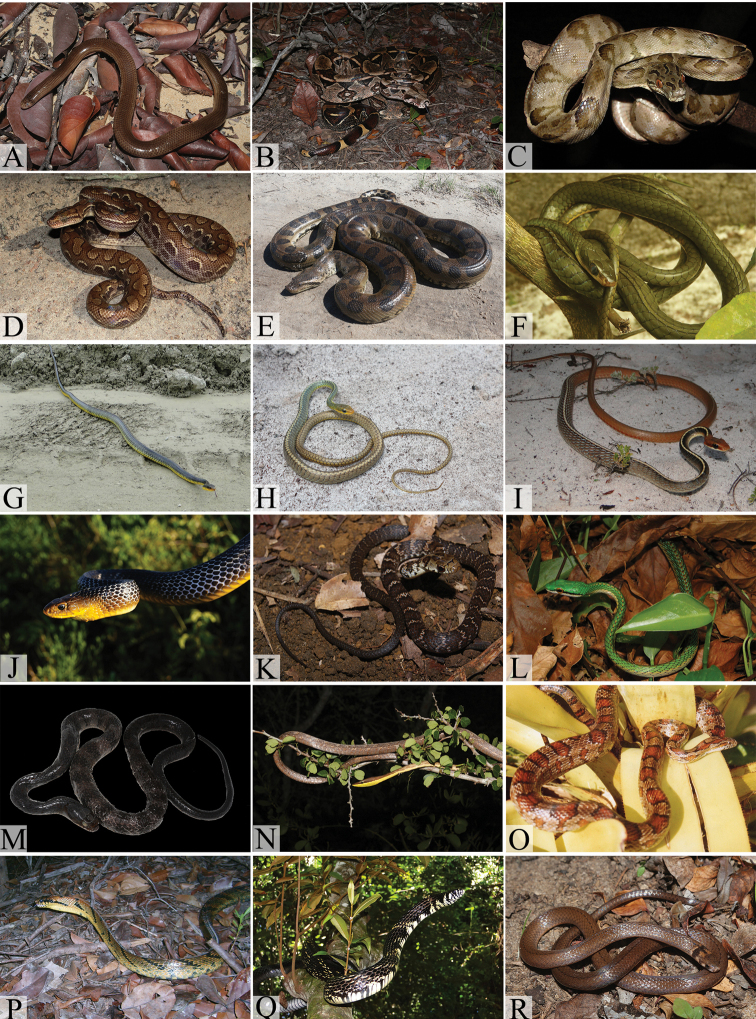
Typhlopid, boids and colubrids from the northern coast of Bahia: **A** *Amerotyphlops brongersmianus* **B** *Boa constrictor constrictor* **C** *Corallus hortulanus* **D** *Epicrates assisi* **E** *Eunectes murinus* **F** *Chironius bicarinatus* **G** *Chironius carinatus* **H** *Chironius exoletus* **I** *Chironius flavolineatus* **J** *Drymarchon corais* **K** *Drymoluber dichrous* (juvenile) **L** *Leptophis ahaetulla liocercus* **M** *Mastigodryas bifossatus* **N** *Oxybelis aeneus* **O** *Pantherophis guttatus* **P** *Pseustes sulphureus sulphureus* **Q** *Spilotes pullatus pullatus* **R** *Tantilla melanocephala*. (Photo **F** by M. A. Passos; **D** by W. Pessoa; **J, K** by M. L. O. Travassos).

#### BOIDAE Gray, 1825

***Boa constrictor constrictor* Linnaeus, 1758**

Fig. 4B

A common species, large sized (min SVL = 430 mm, max SVL = 1850 mm). Recorded at Busca Vida, Camaçari, Imbassaí, Baixio and Costa Azul. Observed from January to November, active from 06:26 h to 21:15 h. We detected adults on ground (n = 8), leaf litter (n = 4), juveniles (SVL ≤ 640 mm) coiled on the base of bromeliad leaves (n = 2) and one specimens each on a fallen trunk, suspended branch, herbaceous vegetation, bushes and anthropogenic material. Inhabits open and forested habitats (Vitt and Vangilder 1983, Vitt et al. 2005), is terrestrial and semiarboreal, active during day and night (Strüssmann and Sazima 1993, this study). Feeds on small mammals, lizards and birds (Pizzatto et al. 2009, Bernarde and Abe 2010). In the municipality of Salvador, a juvenile (SVL ≤ 400 mm) regurgitated an unidentified hummingbird after palpation (pers. obs.). During encounters, behavior of boas ranged from calm to agitated hissing.

***Corallus hortulanus* (Linnaeus, 1758)**

Fig. 4C

A very rare species (see Marques et al. 2012c). It was recorded in an ombrofilous forest remnant on the leaf litter basking in the sun at 14:00 h in September (SVL = 1140 mm). *Corallus hortulanus* is arboreal, often found on vegetation of different habitats during the night (Cunha and Nascimento 1978, Guedes et al. 2014). Feeds on frogs, lizards, birds and small mammals including bats (Henderson et al. 1995, Kok et al. 2006, Pizzatto et al. 2009). We observed this species in an S-coil position, when encountered, followed by strikes and bites when molested.

***Epicrates assisi* Machado, 1945**

Fig. 4D

A single specimen was recorded in the municipality of Mata de São João in ombrophilous forest habitat with a cerrado enclave. After erected to a species level by Passos and Fernandes (2008), the distribution of *Epicrates assisi* is restricted to open habitat of northeastern Brazil but it also apparently colonized disturbed habitats that once were forested (Morato et al. 2011, Garda et al. 2013). It is a nocturnal and terrestrial species (Rodrigues and Prudente 2011) which feeds on small mammals (Pizzatto et al. 2009).

***Eunectes murinus* (Linnaeus, 1758)**

Fig. 4E

A rare species, large sized (min SVL = 1000 mm, max SVL = 3340 mm). Recorded at Praia do Forte, Imbassaí, Massarandupió and Baixio. Due to the aquatic life habit, this species was recorded only on flooded plains in the restinga (n = 3) on May and June, during periods of heavy precipitation. The earliest record was at 07:56 h when the animal was basking among reed vegetation (*Juncus* sp.) in a riverbed, whereas the other observations occurred around 13:00 h. Of those two, one specimen was crossing a road between two lakes and the other was basking in a flooded area among herbaceous vegetation. The species is strongly associated with wetlands, inhabiting rivers and ponds from the Atlantic forest and Amazon, but also moving on land to additionally feed and give birth (Cunha and Nascimento 1978, Argôlo 2004). Feeds on fishes, frogs, lizards, crocodilians, birds and mammals (Beebe 1946, Cunha and Nascimento 1978, Henderson et al. 1995, Martins and Oliveira 1998, Pizzatto et al. 2009).

#### COLUBRIDAE Oppel, 1811

***Chironius bicarinatus* (Wied, 1820)**

Fig. 4F

Dias and Rocha (2014) recorded the species in restinga habitat at Costa Azul. Despite being most frequently encountered in forested habitats and riparian forests, it also occurs in open habitats (Marques and Sazima 2004, Vaz-Silva et al. 2007, Garda et al. 2013). The species is diurnal and forages on soil and in vegetation. It feeds on frogs (Dixon 1993, Marques et al. 2001).

***Chironius carinatus* (Linnaeus, 1758)**

Fig. 4G

A rare species. Recorded at the Instituto da Mata in March and July. Detected in an ombrophilous remnant forest adjacent to a pasture. We observed one large specimen (total length > 1000 mm) swimming in a stream at 16:22 h, while the other record is based on a shed skin. The species inhabits forests, riparian forests, igapós, cocoa plantations, pastures and open habitats. *Chironius carinatus* is diurnal, semiarboreal and terrestrial (Cunha and Nascimento 1978, Vanzolini et al. 1980, Argôlo 2004). It feeds on frogs, birds and lizards (Beebe 1946, Dixon 1993).

***Chironius exoletus* (Linnaeus, 1758)**

Fig. 4H

A very rare species of medium size (min SVL = 190 mm, max SVL = 620 mm). Recorded at Praia do Forte, Imbassaí, Massarandupió and Costa Azul. We observed one specimen foraging on bromeliads in restinga dry forest at 08:33 h in September. Marques et al. (2011) recorded the species on a flooded plain. Voucher specimens originated from restinga (n = 9) and ombrophilous forest (n = 5). *Chironius exoletus* also inhabits forested habitats and pastures. The species is diurnal, terrestrial and semiarboreal, feeding on frogs (Marques and Sazima 2004, Bernarde and Abe 2006, 2010, Hartmann et al. 2009b). Some tried to bite when handled.

***Chironius flavolineatus* (Boettger, 1885)**

Fig. 4I

A common species of medium size (min SVL = 274 mm, max SVL = 1000 mm). Recorded at Busca Vida, Guarajuba, Praia do Forte, Imbassaí, Massarandupió, Baixio and Barra do Itariri. Occurs in the whole region in restinga, ombrophilous forest and urban environments. The species inhabits predominantly open habitats, but also forests, urban and disturbed environments (Carvalho and Nogueira 1998, Vaz-Silva et al. 2007, França et al. 2012, Miranda et al. 2012). Observed from February to December, active from 06:43 h to 17:20 h. We observed specimens on soil (n = 9), in bushes (n = 5), on the leaf litter (n = 3), coiled on a branch during nighttime, in bromeliad, herbaceous vegetation, pond and on a fallen trunk (n = 1 each microhabitat). Feeds on frogs, mainly hylids (Pinto et al. 2008). In Cerrado, females contained oviductual eggs from October to March (Pinto et al. 2010). In September we detected a gravid female (SVL = 610 mm; 3 eggs; 28.5 – 31.2 mm). The species occasionally bites when handled. One specimen was observed to move from leaf litter to suspended branches to evade capture.

***Drymarchon corais corais* (Boie, 1827)**

Fig. 4J

Large sized snake (min SVL = 425 mm, max SVL = 1100 mm). Recorded in the municipalities of Dias D’Ávila and Catu, where vegetation consists mainly of ombrophilous forest. It inhabits the restinga, open areas and urban environments (McCranie 1980, Vaz-Silva et al. 2007, Miranda et al. 2012). The species is diurnal, terrestrial and arboreal and feeds on frogs, lizards, snakes, bird eggs and mammals (Beebe 1946, Cunha and Nascimento 1978, Bernarde and Abe 2010).

***Drymoluber dichrous* (Peters, 1863)**

Fig. 4K

Medium sized snake (min SVL = 220 mm, max SVL = 570 mm). Recorded in the municipality of Catu in ombrophilous forest. It inhabits mainly forested habitats, transition areas and disturbed habitats (Silva et al. 2011, Costa et al. 2013). Diurnal and terrestrial, occasionally resting on vegetation (Martins and Oliveira 1998). It feeds on frogs and lizards (Dixon and Soini 1986, Martins and Oliveira 1998).

***Leptophis ahaetulla liocercus* (Wied, 1824)**

Fig. 4L

A very rare species of medium size (min SVL = 595 mm, max SVL = 850 mm). Recorded at Reserva Sapiranga and Instituto da Mata. We observed a specimen on a suspended branch at 13:22 h in December. Specimens were recorded in restinga dry forests. Inhabits pastures, restinga and urban environment in other regions (Carvalho and Nogueira 1998, Bernarde and Abe 2006, Miranda et al. 2012). Feed mostly on hylid frogs and occasionally on lizards. The species is diurnal and arboreal, foraging in vegetation (Albuquerque et al. 2007, Mesquita et al. 2013). When handled the species intimidates with mouth gaping and bites.

***Mastigodryas bifossatus* (Raddi, 1820)**

Fig. 4M

Medium sized snake (min SVL = 300 mm, max SVL = 802 mm). Recorded in the municipalities of Lauro de Freitas, Mata de São João, Catu and Jandaíra, without information on habitat. Terrestrial and diurnal, inhabits forested and deforested environments (Strüssmann and Sazima 1993). Argôlo (2004) and Leite et al. (2007) observed most specimens in plantation and around human habitation. Feeds preferably on frogs, also lizards, snakes, birds and mammals (Strüssmann and Sazima 1993, Leite et al. 2007, Marques and Muriel 2007).

***Oxybelis aeneus* (Wagler, 1824)**

Fig. 4N

An intermediate frequent species of medium size (min SVL= 236 mm, max SVL = 754 mm). Recorded at Arembepe, Praia do Forte and Imbassaí from June to November. Found in activity between 09:05 h and 12:45 h. We observed individuals on suspended branches (n = 2) and bush (n = 1). In the region, it inhabits restinga and ombrophilous forest. In other ecoregions, it occupies also primary forests, disturbed and open habitats (Keiser 1982, Silva et al. 2011, Miranda et al. 2012, Garda et al. 2013). The species is arboreal and diurnal, feeding mostly on lizards and occasionally frogs (Martins and Oliveira 1998, Marques et al. 2001). We observed mouth gaping or immediate fleeing.

***Spilotes pullatus pullatus* (Linnaeus, 1758)**

Fig. 4P

A rare species of large size (in SVL = 139.1 mm, max SVL = 1600 mm). Recorded at Busca Vida, Praia do Forte and Imbassaí in July and August. We observed an individual foraging on the leaf litter at 15:46 h. It inhabits forests, open and disturbed habitats, is diurnal and semiarboreal (Vanzolini et al. 1980, Argôlo 2004, Bernarde and Abe 2006). It feeds on frogs, lizards, birds, eggs, marsupials and rodents (Cunha and Nascimento 1978, Marques and Sazima 2004). We observed immediate fleeing or inflating of the gular region.

***Spilotes sulphureus sulphureus* (Wagler, 1824)**

Fig. 4Q

A very rare species. Recorded at Praia do Forte in August. Marques et al. (2011) reported the species from Imbassaí, captured in a restinga dry forest (SVL = 1970 mm). Inhabits primary forest, ombrophilous forest, relict wet forest and cabruca, foraging more on vegetation than on the ground (Martins and Oliveira 1998, Argôlo 2004, Lisboa et al. 2009, Loebmann and Haddad 2010). The species is arboreal and diurnal, feeding on birds and rodents (Beebe 1946, Marques et al. 2001). We observed defensive behaviors of elevating the anterior of the body, striking and biting.

***Tantilla melanocephala* (Linnaeus, 1758)**

Fig. 4R

A rare species of small size (min SVL = 130 mm, max SVL = 277 mm). Recorded at Busca Vida, Praia do Forte and Imbassaí. Occurred in restinga (n = 9) and ombrophilous forest (n = 2). The recorded specimens were active at 06:00 h and 19:00 h in May and June, both foraging on soil. The species is terrestrial, fossorial, diurnal and nocturnal (Vanzolini et al. 1980, Marques and Puorto 1998, Sawaya et al. 2008, this study). It feeds on insects and mostly chilopods. Santos-Costa et al. (2006) report ovoposition of three eggs in February, June and September. We observed the ovoposition of two eggs in May (SVL = 277 mm). The tail of this species apparently breaks easily, since we observed two specimens from different sites with broken tails near the cloaca, not exceeding eight subcaudal scales.

#### DIPSADIDAE Bonaparte, 1838

***Sibynomorphus neuwiedi* (Ihering, 1911)**

Fig. 5A

A rare species of small size (min SVL = 175 mm, max SVL = 455 mm). Recorded at Instituto da Mata in July and October and the municipalities of Pojuca and Catu. Dias and Rocha (2014) reported this species from the restinga of Costa Azul. We found this species only in ombrophilous forest. It is nocturnal, terrestrial and semiarboreal and feeds exclusively on slugs (Maia-Carneiro et al. 2012). Females from southeastern Brazil exhibited vitellogenesis from July to December and oviductual eggs from August to February (Pizzatto et al. 2008). We observed seven ovarian follicles (3.6 – 4.7 mm) in October and oviductual eggs (SVL = 480 mm; 6 eggs; 14.5 – 16.6 mm) in April. If threatened, it flattens its head.

**Figure 5.**
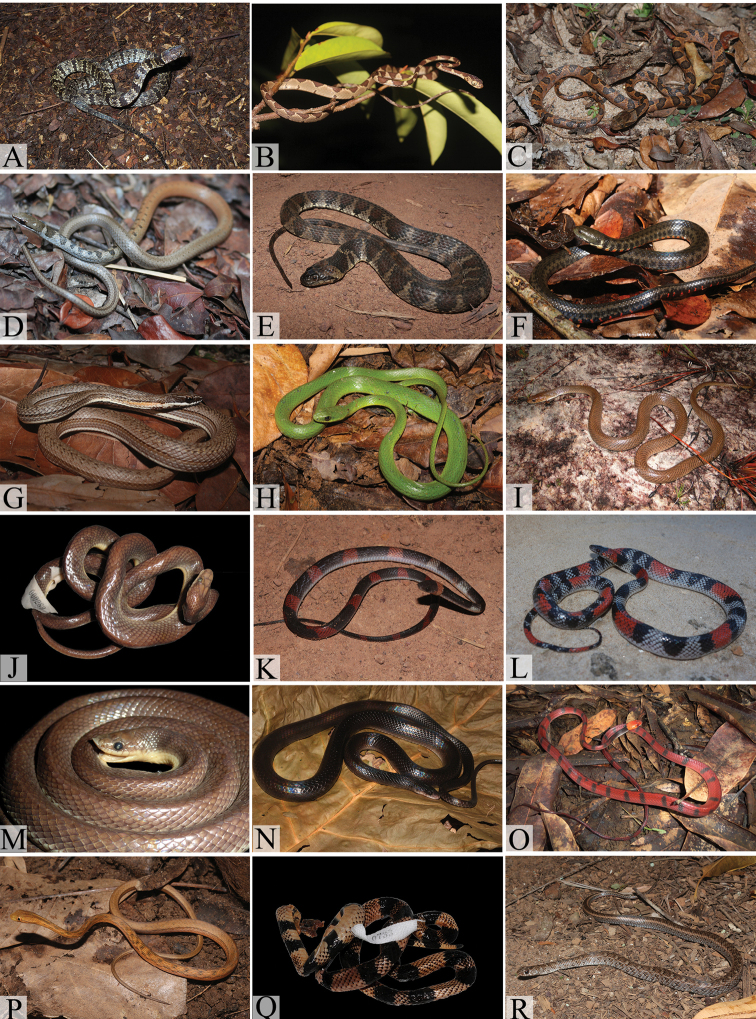
Dipsadids from the northern coast of Bahia: **A** *Sibynomorphus neuwiedi* **B** *Imantodes cenchoa cenchoa* **C** *Leptodeira annulata annulata* **D** *Taeniophallus occipitalis* **E** *Helicops angulatus* **F** *Helicops leopardinus* **G** *Philodryas nattereri* **H** *Philodryas olfersii herbeus* **I** *Philodryas patagoniensis* **J** *Clelia plumbea* **K** *Oxyrhopus petolarius digitalis* **L** *Oxyrhopus trigeminus* **M** *Phimophis guerini* **N** *Pseudoboa nigra* **O** *Siphlophis compressus* **P** *Thamnodynastes pallidus* **Q** *Erythrolamprus aesculapii venustissimus* **R** *Erythrolamprus almadensis*.

***Imantodes cenchoa cenchoa* (Linnaeus, 1758)**

Fig. 5B

A very rare species of medium size (min SVL = 455 mm, max SVL = 670 mm). Recorded at Instituto da Mata in November (see Marques et al. 2013) and the municipality of Simões Filho. Occurs in the ombrophilous forest in the study region. The species is nocturnal and we recorded it at 20:23 h. The animal was foraging on a bromeliad. Despite its arboreal habit, *Imantodes cenchoa* often forages on ground level in forests and disturbed habitats (Cunha and Nascimento 1978). It feeds on frogs and lizards (Marques et al. 2001). Cloacal discharge was observed.

***Leptodeira annulata annulata* (Linnaeus, 1758)**

Fig. 5C

An intermediately frequent species of medium size (min SVL = 195 mm, max SVL = 325 mm). Recorded at Praia do Forte, Reserva Sapiranga, Imbassaí, Massarandupió, Baixio and Costa Azul from May to October. Occurs in wooded vegetation of restinga and ombrophilous forest. Also inhabits forests, open and dry habitats (Martins and Oliveira 1998, Garda et al. 2013). The species is nocturnal and we found it active from 18:05 h to 21:00 h. The snakes were observed on soil (n = 3), in anthropogenic environment (n = 1) and two meters high on vegetation (n = 1), always near ponds or lakes. It feeds on frogs, lizards and snakes (Cantor and Pizzatto 2008). Head flattening and cloacal discharge was observed.

***Taeniophallus occipitalis* (Jan, 1863)**

Fig. 5D

An intermediately frequent species of small size (min SVL = 215 mm, max SVL = 325 mm). Recorded at Praia do Forte, Instituto da Mata and Reserva Sapiranga from May to November. Occurs in restinga (n = 4), disturbed ombrophilous forest (n = 1) and pasture (n = 1). Also distributed in the Amazon and Caatinga (Argôlo 2004). The species is diurnal, active from 08:47 h to 10:20 h (n = 3) and one record at 16:48 h. Strictly terrestrial, we observed the species on the leaf litter (n = 4). It feeds on frogs, lizards and snakes (Cunha and Nascimento 1978, Argôlo 2004, Balestrin and Di-Bernardo 2005). A female contained oviductal eggs (SVL = 315 mm; 3 eggs; ≤ 12.5 mm) in May.

***Helicops angulatus* (Linnaeus, 1758)**

Fig. 5E

An intermediately frequent species of medium size (min SVL = 173 mm, max SVL = 810 mm). Recorded at Praia do Forte, Reserva Sapiranga, Instituto da Mata and Costa Azul in March and from September to November. Occurs in ombrophilous forest (n = 10) and restinga (n = 3). The species can be abundant in urbanized and open environments and less frequent in forest habitats (Martins and Oliveira 1998, França et al. 2012). We observed snakes in rain puddles, lake shores and rivers and in open areas. *Helicops angulatus* is mainly nocturnal, active from 18:38 h to 21:08 h (n = 10) with a single record at 10:42. It feeds on fishes and frogs (Dixon and Soini 1986, Martins and Oliveira 1998, Ford and Ford 2002). We observed defensive behaviors, such as biting, struggling and occasional cloacal discharge.

***Helicops leopardinus* (Schlegel, 1837)**

Fig. 5F

A very rare species of medium size (min SVL = 109 mm, max SVL = 638 mm). Recorded at Arembepe, Imbassaí and Baixio in February and October. We found only specimens in the restinga, but vouchers originate also from forested habitats (n = 13). We observed snakes at edges of lakes and only during diurnal activity from 10:00 h to 16:12 h. However, *Helicops leopardinus* is known to be mainly nocturnal. It feeds on fishes and frogs (Strüssmann and Sazima 1993, Ávila et al. 2006). We observed biting when handled.

***Philodryas nattereri* Steindachner, 1870**

Fig. 5G

A common species of medium size (min SVL = 295 mm, max SVL = 840 mm). Recorded at Arembepe, Guarajuba, Praia do Forte, Imbassaí, Massarandupió, Baixio, Barra do Itariri and Costa Azul during the year. We found it only in restinga (n = 38) during fieldwork, supported also by voucher specimens. It inhabits mainly open and semiarid habitats, also recorded at brejos de altitude (isolated fragments of humid forests surrounded by arid Caatinga) and disturbed habitats (Vanzolini et al. 1980, Marques et al. 2011, Pereira-Filho and Montingelli 2011, Mesquita et al. 2013, this study). The species is diurnal and active from 08:30 h to 17:50 h. *Philodryas nattereri* is mainly terrestrial, as we observed it on the ground (n = 19), on the leaf litter (n = 14), among bush branches (n = 2), over herbaceous vegetation (n = 1) and on a branch that was four meters suspended (n = 1). It feeds on frogs, lizards, snakes, birds and mammals (Mesquita et al. 2011). We observed the elevating of its anterior body and immediate fleeing behavior, where it moved from the ground to a nearby suspended branch.

***Philodryas olfersii herbeus* Wied, 1825**

Fig. 5H

An intermediately frequent species of medium size (min SVL = 200 mm, max SVL = 770 mm). Recorded at Busca Vida, Arembepe, Instituto da Mata, Imbassaí, Massarandupió and Costa Azul in January and from July to December. We found it in restinga (n = 9) and disturbed areas of ombrophilous forest (n = 2). The species is diurnal and active from 07:56 h to 14:56 h. We observed snakes foraging on ground (n = 3), on leaf litter (n = 2) and in herbaceous vegetation (n = 1). The species is terrestrial and semiarboreal (Marques et al. 2001). It feeds on frogs, birds and rodents (Leite et al. 2009). In the Caatinga domain, the species is mating from November to January (Mesquita et al. 2013). We analyzed a fertile male (SVL = 551 mm) in November. We observed cloacal discharge and biting as a defensive behaviour.

***Philodryas patagoniensis* (Girard, 1858)**

Fig. 5I

A common species of medium size (min SVL = 184 mm, max SVL = 848 mm). Recorded at Busca Vida, Arembepe, Praia do Forte, Imbassaí, Massarandupió, Barra do Itariri and Costa Azul from February to December. Occurs in restinga (n = 14), urbanized environment (n = 5) and ombrophilous forest (n = 3). Usually found in open and disturbed habitats (Sawaya et al. 2008, Hartmann et al. 2009a). The species is diurnal, active from 06:54 h to 15:48 h. It is terrestrial and we observed it on the ground (n = 8), on leaf litter (n = 4) and among bushes (n = 1). It feeds on frogs, lizards, snakes, birds and small mammals (Hartmann and Marques 2005). Marques et al. (2012a) observed this species predating on *Philodryas olfersii*.

***Clelia plumbea* (Wied, 1820)**

Fig. 5J

A medium sized snake (min SVL = 335 mm, max SVL = 820 mm). Recorded in the municipality of Catu. Voucher specimens were from urban habitation. Inhabits mainly forests, disturbed, and open habitats (Zaher 1996, Argôlo 2004). The species is terrestrial and diurnal, feeding on lizards, snakes and mammals (Bernarde and Abe 2006). The reproductive cycle is continuous and the clutch size varies from four to 29 eggs (Gaiarsa et al. 2013).

***Oxyrhopus petolarius digitalis* Reuss, 1834**

Fig. 5K

A rare species of small size (min SVL = 176 mm, max SVL = 445 mm). Recorded at Praia do Forte and Instituto da Mata in July and November. We observed animals on pasture (n = 2) near ombrophilous forest remnants. A voucher specimen was obtained from the restinga. The species is terrestrial, forages on the ground (n = 2) and is nocturnal, detected at 21:10 h. Inhabits forest and disturbed habitats (Argôlo 2004, França et al. 2012). It feeds on lizards, birds, eggs and mammals and has a clutch size of two to 12 eggs (Gaiarsa et al. 2013). We observed winding when handled.

***Oxyrhopus trigeminus* Duméril, Bibron & Duméril, 1854**

Fig. 5L

An intermediately frequent species of medium size (min SVL = 178 mm, max SVL = 656 mm). Recorded at Busca Vida, Arembepe, Guarajuba, Praia do Forte, Imbassaí, Massarandupió, Baixio, Barra do Itariri and Costa Azul from January to October. Occurs in restinga, ombrophilous forest and anthropic environments. The species is mainly nocturnal and we observed it from 18:05 h to 21:00 h, except for two specimens foraging at 09:50 h and 11:00 h. *Oxyrhopus trigeminus* is terrestrial. It was recorded on the ground (n = 5), in the leaf litter (n = 2), in bush and herbaceous vegetation (n = 1 each). It feeds on lizards, birds and small mammals. In southeastern Brazil, females are fertile from January to November (Alencar et al. 2012). We observed two reproductive males (SVL = 380 and 440 mm) and a female with ovarian follicles (SVL = 450 mm; n = 6; ≥ 3 mm) in May. Defensive behaviour consists of occasional cloacal discharge.

***Phimophis guerini* (Duméril, Bibron & Duméril, 1854)**

Fig. 5M

A medium sized snake (min SVL = 323 mm, max SVL = 754 mm). Recorded at Guarajuba, Praia do Forte and Imbassaí (see Marques et al. 2012b). Occurs in restinga (n = 3) and anthropogenic habitat (n = 1). Dias and Rocha (2014) report the species from the restinga of Costa Azul. This species inhabits several types of open habitat including urban environments (Carvalho and Nogueira 1998, Sawaya et al. 2008, Valdujo et al. 2009). It feeds on lizards (Sawaya et al. 2008). Gaiarsa et al. (2013) reports clutch size of three to seven eggs.

***Pseudoboa nigra* (Duméril, Bibron & Duméril, 1854)**

Fig. 5N

A medium sized snake (min SVL = 214 mm, max SVL = 870 mm). Recorded at Busca Vida and Jauá, and in the municipalities of Mata de São João and Catu. Occurs in ombrophilous forests and restinga. *Pseudoboa nigra* also inhabits open and disturbed habitats and forests (Argôlo 2004, Gaiarsa et al. 2013). The species is terrestrial and nocturnal. It feeds on frogs, lizards and mammals (Vanzolini et al. 1980, Vitt and Vanglinder 1983, Orofino et al. 2010, França et al. 2012). Orofino et al. (2010) reported offspring with a SVL of 340 mm. We analyzed offspring with SVL of 214 mm and 274 mm.

***Siphlophis compressus* (Daudin, 1803)**

Fig. 5O

A very rare species of medium size (SVL = 738 mm). Recorded at Instituto da Mata (see Marques et al. 2013) in May. We found it in ombrophilous forest remnants foraging at ground level on the leaf litter at 21:00 h. The species inhabits forests in Amazonia and Atlantic coast (Guedes et al. 2011). Nocturnal and arboreal, occasionally found on the ground. It feeds mainly on lizards (Martins and Oliveira 1998, Marques et al. 2001, Argôlo 2004). Clutch size varies from 3–12 eggs (Gaiarsa et al. 2013).

***Thamnodynastes pallidus* (Linnaeus, 1758)**

Fig. 5P

A very rare species of small size (SVL = 363 mm). Recorded at the Instituto da Mata (see Marques et al. 2013). We observed it in ombrophilous forest remnants. We confirm that this snake is nocturnal, as we found one foraging on the ground at 20:30 h. The female did not contain ovarian follicles in May. Marques et al. (2014) report ovarian follicles in July and eggs in September. Cunha and Nascimento (1978) report *Thamnodynastes pallidus* inhabiting the humid ground on forests and feeding on frogs and insect larvae.

***Erythrolamprus aesculapii venustissimus* Wied, 1821**

Fig. 5Q

A medium sized snake (SVL = 655 mm). Recorded in the municipalities of Lauro de Freitas collected in 1994, without habitat information. Morato et al. (2011) record the species from the state of Sergipe at 175 km from our record. Inhabits forests, disturbed habitats and plantations (Sazima and Abe 1991, Argôlo 2004). This species is terrestrial and diurnal, but occasionally nocturnal, feeding mainly on snakes (Sazima and Abe 1991, Marques and Sazima 2004).

***Erythrolamprus almadensis* (Wagler, 1824)**

Fig. 5R

Rare species of medium size (Min SVL = 136 mm, Max SVL = 450 mm). Recorded at Arembepe, Instituto da Mata, Imbassaí and Baixio in March and October. Occurs in ombrophilous forest (n = 8), restinga (n = 4), pasture and disturbed habitats (n = 1 each). Also recorded from urban environment (França et al. 2012). We recorded diurnal activity at 08:21 h and 11:40 h. The species is terrestrial and feeds on frogs (Strüssmann and Sazima 1993, Bernarde and Abe 2010).

***Erythrolamprus miliaris merremi* (Wied, 1821)**

Fig. 6A

A medium sized snake (min SVL = 145, max SVL = 642 mm). Recorded from the municipalities of Simões Filho, Dias D’Ávila and Pojuca. Occurs in ombrophilous forest remnants and disturbed areas. The species is semiaquatic with diurnal and nocturnal activities, inhabiting forests and open habitats. It feeds on fishes, caecilians, frogs, amphisbaenians, lizards, and snakes (Marques and Sazima 1994, Bonfiglio and Lema 2006, Hartmann et al. 2009b).

**Figure 6.**
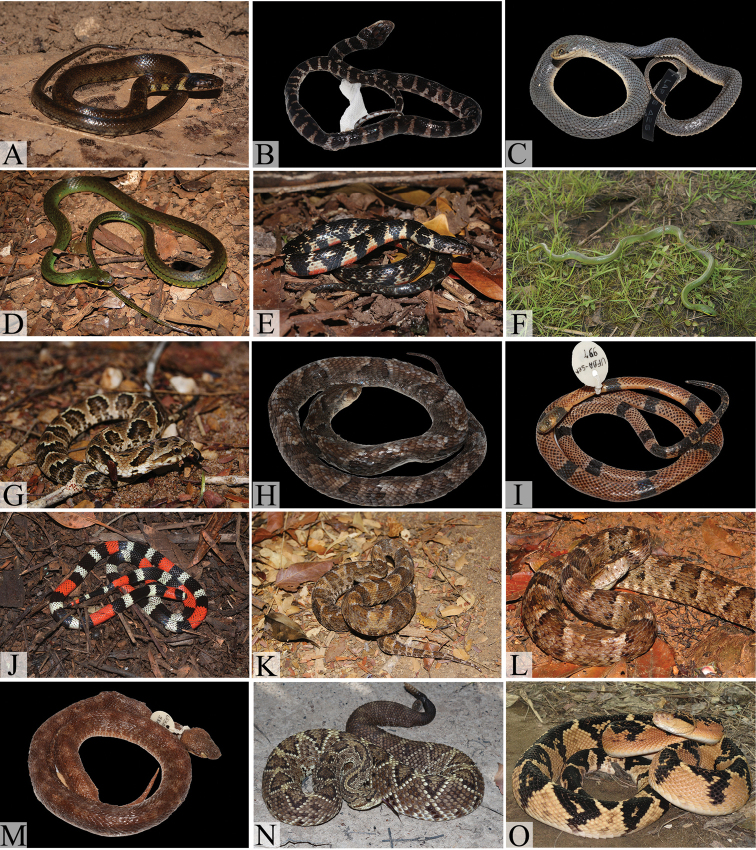
Dipsadids, elapids and viperids from north coast of Bahia: **A** *Erythrolamprus miliaris merremi* **B** *Erythrolamprus poeilogyrus schotti* (juvenile with a *Erythrolamprus poeilogyrus poecilogyrus*-like pattern) **C** *Erythrolamprus poecilogyrus schotti* (adult) **D** *Erythrolamprus reginae semilineatus*, **E** *Erythrolamprus taeniogaster* **F** *Erythrolamprus viridis viridis* **G** *Xenodon merremii* **H** *Xenodon rhabdocephalus rhabdocephalus* **I** *Micrurus corallinus* **J** *Micrurus ibiboboca* **K** *Bothrops erythromelas* **L** *Bothrops leucurus* **M** *Bothrops lutzi* **N** *Crotalus durissus cascavella* **O** *Lachesis muta rhombeata* (Photos **F, G, K** by M.L.O. Travassos; **O** by W. Pessoa).

***Erythrolamprus poecilogyrus schotti* (Schlegel, 1837)**

Fig. 6B, C

A medium sized snake (min SVL = 150 mm, max SVL = 623 mm). Recorded from the municipalities of Camaçari, Lauro de Freitas, Mata de São João, Catu and Pojuca. It occurs in ombrophilous forest, but inhabits also cocoa plantations, pastures, swamps, and urbanized habitats (Pontes and Rocha 2008). One specimen was obtained from an industrial complex. In the state of Bahia, it occurs in the area north of Belmonte, being replaced by the nominate taxon *Erythrolamprus poeilogyrus poecilogyrus* farther south (Fernandes 2006). The species is diurnal and nocturnal, and feeds on frogs and lizards (Marques et al. 2001, Argôlo 2004, Sawaya et al. 2008). Populations from the Atlantic forest from northeastern Brazil reproduce throughout the year with a clutch size of 3–17 eggs (Pizzatto et al. 2008b).

***Erythrolamprus reginae semilineatus* (Linnaeus, 1758)**

Fig. 6D

A very rare species of small size (min SVL = 191 mm, max SVL = 494 mm). Recorded at the Instituto da Mata (see Marques et al. 2013) and the municipalities of Catu and Pojuca. We observed it in ombrophilous forest in February. Commonly found near ponds, forest edges, fields and disturbed habitats (Dixon and Soini 1986). The snake was foraging on the leaf litter at 10:26 h. The species is terrestrial, occasionally using the vegetation to rest (Martins and Oliveira 1998). It feeds on frogs, tadpoles and lizards (Cunha and Nascimento 1978, Bernarde and Abe 2010). We detected a gravid female (SVL = 403 mm; 1 egg, 26 mm) in June. We observed dorsoventral flattening.

***Erythrolamprus taeniogaster* Jan, 1863**

Fig. 6E

A very rare species of medium size (min SVL = 147 mm, max SVL = 583 mm). Recorded at Praia do Forte, Imbassaí, and the municipalities of Lauro de Freitas, Simões Filho, Camaçari and Catu. We observed it in restinga in June, with voucher specimens also from restinga (n = 5) and ombrophilous forest (n = 4). In northeastern and southeastern Brazil, the species has a coastal distribution (Fernandes et al. 2002). In southern Bahia, Argôlo (2004) reported the species from sites near flooded areas, dams and swamps. We observed the species on a dirt road near a flooded plain at 15:00 h in urban habitation. The species struggles when handled.

***Erythrolamprus viridis viridis* (Günther, 1862)**

Fig. 6F

A small sized snake (SVL = 382 mm). Recorded in the municipality of Mata de São João. Occurred in ombrophilous forest, but also in transition areas from Atlantic forests and agreste (hilly north south chain with hot and sub-humid climate) region (Dixon 1987). The species is terrestrial but forages also in vegetation (Vanzolini et al. 1980). It feeds mainly on frogs and lizards (Vitt 1983, Mesquita et al. 2013).

***Xenodon merremii* (Wagler, 1824)**

Fig. 6G

A very rare species of large size (min SVL = 186 mm, max SVL = 1003 mm). Recorded at Guarajuba, Praia do Forte, Instituto da Mata, Imbassaí and Sauípe. Occurs in open areas. Voucher specimens were from deforested ombrophilous forest (n = 9) and restinga (n = 6). Terrestrial and diurnal, feeds exclusively on amphibians (Vitt 1983, Vitt and Vanglinder 1983). Pizzatto et al. (2008b) report offspring of *Xenodon merremii* between January and May in southeastern Brazil. We captured one offspring in July.

***Xenodon rabdocephalus rabdocephalus* (Wied, 1824)**

Fig. 6H

A medium sized snake (min SVL = 232 mm, max SVL = 654 mm). Recorded from the municipalities of Camaçari and Catu. Occurs in ombrophilous forest, cocoa plantation and pastures. The species is terrestrial and feeds on frogs and occasionally on tadpoles (Dixon and Soini 1986, Martins and Oliveira 1998, Argôlo 2004).

#### ELAPIDAE Boie, 1827

***Micrurus corallinus* (Merrem, 1820)**

Fig. 6I

A small sized snake (SVL= 405 mm). Recorded from the municipality of Simões Filho in ombrophilous forest. Campbell and Lamar (2004) report this species from forested areas with coastal (maritime) influence. *Micrurus corallinus* is diurnal and cryptozoic, feeding on frogs, amphisbaenians and snakes (Lema et al. 1983, Marques and Sazima 1997, Marques and Sazima 2004).

***Micrurus ibiboboca* (Merrem, 1820)**

Fig. 6J

A common species of medium size (min SVL= 210 mm, max SVL = 1092 mm). Recorded at Busca Vida, Arembepe, Jacuípe, Guarajuba, Praia do Forte, Reserva Sapiranga, Instituto da Mata, Imbassaí, Sauípe, Baixio and Barra do Itariri from January to November. We observed it in restinga (n = 9), ombrophilous forest (n = 2), deforested, open and disturbed habitats (n = 3). In restinga, it inhabits the dry part of the forest. We found it active on the leaf litter (n = 9) and soil (n = 5) from 08:11 h to 17:30 h. *Micrurus ibiboboca* is ophiophagous (Lema et al. 1983, Marques et al. 2001, Campbell and Lamar 2004). We captured a reproductive male in November (SVL = 520 mm). Defensive or protective behaviour included immediate fleeing, coiling and elevating the tail, dorsoventral flattening, head hiding under its body and struggling when handled.

#### VIPERIDAE Oppel, 1811

***Bothrops erythromelas* Amaral, 1923**

Fig. 6K

A medium sized snake (adult SVL ca. 570 mm). Recorded from the municipalities of Camaçari and Lauro de Freitas (MZUFBA 499 and 1366, respectively). The vegetation of both municipalities varies from restinga to ombrophilous forest with enclaves of cerrado, but no precise information was available on either of the two specimens' habitats. This is a terrestrial and nocturnal species that inhabits arid and semiarid regions, from sandy and rocky areas to deciduous forests, mainly associated with the Caatinga and Cerrado (Campbell and Lamar 2004).

***Bothrops leucurus* Wagler, 1824**

Fig. 6L

An intermediately frequent species of large size (min SVL = 115 mm, max SVL = 1600 mm). Recorded at Arembepe, Guarajuba, Itacimirim, Praia do Forte, Instituto da Mata, Imbassaí, Sauípe, Massarandupió, Baixio and Mangue Seco. We found it in restinga (n = 9), nearby human habitation and pastures (n = 1) from February to October. *Bothrops leucurus* is more active during nighttime (Argôlo 2004), but we observed it mainly active from 06:10 h to 08:15 h. One adult moved at 15:30 h and two juveniles at 20:00 h and 20:08 h. We observed it mostly on the ground (n = 5), moving over bushes, coiled on short palm trees and on anthropogenic material (n = 1 each). Juveniles feed on frogs and adults mostly on rodents (Argôlo 2004, Fagundes et al. 2009). When threatened, they coil their body, vibrate the tail and occasionally strike.

***Bothrops lutzi* (Miranda-Ribeiro, 1915)**

Fig. 6M

A small sized snake (SVL = 451 mm). This species is confirmed from north of Salvador by four specimens, three by Lira-da-Silva et al. (2003) near sea level in the municipalities of Camaçari and Dias D’Ávila and one voucher at the Institute Butantan, also from Camaçari (IBSP 959). Terrestrial species, inhabits savannas and higher plateaus in the Cerrado (Loebmann 2009, Campbell and Lamar 2004) and Caatinga < 250 m asl. (Loebmann and Haddad 2010).

***Crotalus durissus cascavella* Wagler, 1824**

Fig. 6N

A rare species of medium size (min SVL = 255 mm, max SVL = 1268 mm). Recorded at Busca Vida, Imbassaí and Massarandupió on March, August and September. Voucher specimens were from ombrophilous forest (n = 9), probably from deforested areas (Bastos et al. 2005) and restinga (n = 4). We observed a specimen crossing a road at 20:00 h while others were found on soil and leaf litter. This species is terrestrial and nocturnal, inhabiting open habitats and feeding on rodents (Vanzolini et al. 1980, Graça Salomão et al. 1995, Tozetti and Martins 2013). When handled the squirting of cloacal discharge can reach 1.5 m and if threatened, they coil their body and vibrate the tail and rattle.

***Lachesis muta rhombeata* Wied-Neuwied, 1824**

Fig. 6O

A single record exists from the region based on a skin of an animal captured on a farm in the municipality of Entre Rios in 1996. The species inhabits primary forests, riparian forest, lowlands and cocoa plantations in southern Bahia. Occasionally it is found in secondary forest and surroundings. The species is nocturnal and feeds on small rodents (Cunha and Nascimento 1978, Argôlo 2004).

## Discussion

The number of snake species from the northern coast of Bahia represents 38% of all species known from the state of Bahia (Hamdan and Lira-da-Silva 2012, Curcio et al. 2012, Fernandes and Hamdan 2014) and contains six families. Disregarding the mistaken record of *Bothrops jararaca* by Marques et al. (2011), we recorded 49 native species in the study region. The absence of 17 species during our fieldwork, confirmed only from older museum specimens without new voucher deposits over at least ten years, suggests they have either become extinct in the region or our search method was insufficient (time spent) or non-appropriate, since our larger effort were on restinga. Three of these missing species (*Drymarchon corais corais*, *Phimophis guerini* and *Pseudoboa nigra*) are known from restinga habitat, whereas other species relate mostly to wooded areas, such as *Drymoluber dichrous*, *Clelia plumbea*, *Erythrolamprus aesculapii venustissimus*, *Erythrolamprus miliaris merremii*, *Erythrolamprus viridis viridis*, *Xenodon rhabdocephalus rhabdocephalus*, *Micrurus corallinus* and *Lachesis muta rhombeata*. These assumptions can only be confirmed with additional sampling efforts in the few forest remnants of the region.

Three species require taxonomic comments. First, the pitviper *Bothrops lutzi* from our study region, which appears isolated and far distant from the next known populations of ca. 425 km distance in Petrolina, Pernambuco, which was erroneously reported as 325 km in Lira-da-Silva (2000). We measured three specimens from Camaçari and Dias d’Ávila with ventral scale counts from 153–156, confirming Campbell and Lamar's (2004) findings that these are the lowest scale counts known for this species, which usually ranges from 161–179 ventral scales. These low values are more typical for *Bothrops erythromelas* which exhibits 160 or lower ventral scales, which raises the question of potential mis-identification or hybridization between both viperid species. However, the *Bothrops erythromelas* from the same region north of Salvador (Camaçari and Lauro de Freitas) yielded species typical 19-19-17 dorsal scales, whereas the sympatric *Bothrops lutzi* exhibited species specific 21/23-21-19 dorsal scales. Moreover, the color pattern of latter specimens from Camaçari and Dias D’Ávila resembles rather typical *Bothrops lutzi* than *Bothrops erythromelas*, including diffuse and narrow trapezoid dorsal blotches (approaching a quadrangle), a diffuse and posteriorly not broadening postorbital stripe beginning behind the eye, and labials mostly darkened or mottled in the dorsum ground color with a few white spots. In contrast, *Bothrops erythromelas* has comparatively wider angled dorsal blotches (resembling triangles, either pointed or rounded at the top), a larger postorbital stripe beginning below the eye and broadening posteriorly, and labials mostly white with occasional few larger spots. Machado et al. (2014) reported shared haplotypes between the two pitvipers, suggesting an introgressive hybridization, where the two species come into contact in western Bahia. The low ventral counts for the *Bothrops lutzi* from the municipality of Salvador region may be another example of hybridization/introgression, but so far this is only suggested due to the low ventral scale count, which alternatively may represent a case of geographically isolated variation.

**Figure 7.**
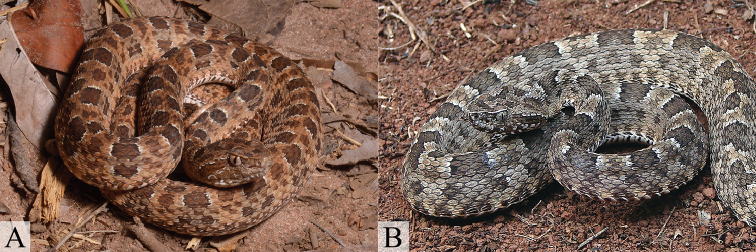
Strongly marked polymorphism of *Bothrops lutzi* from São Desidério, Bahia (**A**) and Grande Sertão Veredas National Park, Minas Gerais (**B**) (Photos **A** by R. Gaiga; **B** by M. Sacramento).

Furthermore, there are several enclaves of Cerrado type vegetation (Campo Cerrado, Campo-limpo-de-Cerrado) from Camaçari (Salvador region) 200 km north to Ribeiro do Pombal, Bahia (Ab’Saber 1977, IBGE 2004). These enclaves allow the unusual presence of “Cerrado-species” in this area, such as *Dendropsophus nanus*, *Dendropsophus rubicundulus*, *Hypsiboas raniceps* and *Dermatonotus muelleri* (Xavier et al. 2015). These remnant patches of Cerrado vegetation propose a scenario of a previous grassland corridor for an expansion of *Bothrops lutzi* from its western populations in the current Cerrado domain (approx. 450 km distant). Subsequent aridification and expansion of Caatinga domain interrupted the corridor (Ab’Saber 1977) with the populations north of Salvador becoming relictual during the late Pleistocene 0.63 – 0.11 mya (Ab’Saber 1977, Prado and Gibbs 1993, Pennington et al. 2000, Werneck 2011, Machado et al. 2014), while the intervening dried area was colonized by *Bothrops erythromelas*. Drastic urban sprawl and intense agricultural practices may have also extirpated local grassland populations of *Bothrops lutzi*. This may relate to notes that *Bothrops lutzi* was once common in the low and humid region of southern Rio Paraguҫu, Recôncavo da Bahia, close to Salvador (*Bothrops lutzi* labelled as *Bothrops neuwiedi neuwiedi* in Amaral 1925, see cit. in Silva and Rodrigues 2008). Possibly more relictual *Bothrops lutzi* populations exist on higher elevated grassland within the Caatinga today. For example, the holotype of *Bothrops neuwiedi bahiensis* Amaral, 1925 (IBSP 3012) originates from Itiuba, Bahia, a town in the midst of mountains that might have yielded grassland suitable for *Bothrops lutzi*, while it is surrounded by Caatinga, that likely provides habitat for *Bothrops erythromelas*. Similarly, combined information suggests sympatry of these vipers in central Piauí (Cambell and Lamar 2004, Benicio et al. 2015) or Guanambi (Silva and Rodrigues 2008, Machado et al. 2014). The specimen from Itiuba is perceived as *Bothrops neuwiedi* by Silva and Rodrigues (2008, and their figs 14A–B), but possibly represents a *Bothrops lutzi* based on the authors' qualitative characters assigned to *Bothrops lutzi*, such as diffuse, at least partialy, interspace blotches, white supralabial spots vertically directed on 4^th^ and posterior supralabials, white spotting more marked posterior the 3^rd^ supralabial, and lack of dorsal postcephalic stripe. In contrast, this specimen has also well defined dorsal blotches, a neuwiedi character acc. Silva and Rodrigues (2008). However, the first author of the latter publication co-authored new records of *Bothrops lutzi* from Minas Gerais (Moura et al. 2013), that, together with records of an adult *Bothrops lutzi* from São Desidério in Bahia or a juvenile of approximately 30 cm total length from Grande Sertão Veredas National Park, Minas Gerais (Figure 7), indicate that these *neuwiedi*-characters are often within the variation of *Bothrops lutzi*, rendering it more polymorphic than previously described. The suggestion of *Bothrops lutzi* at Itiuba is also biogeographically plausible, as this specimen is nearest to other *Bothrops lutzi*, north at Petrolina, Paraíba (Silva and Rodrigues 2008) southeast at Camaçari and Dias d’Avila (Lira-da-Silva et al. 2003) and west at Ibiraba, Bahia (Machado et al. 2014).

Comments on the second species relate to the confusingly polymorphic species *Erythrolamprus poecilogyrus* divided into multiple subspecies based mainly on color pattern variation that shows also drastic changes throughout ontogeny. Offspring and juvenile pattern resembling two taxa, *Erythrolamprus poecilogyrus poecilogyrus* and *Erythrolamprus poecilogyrus schotti*, have been recorded by us from the study area. According to Dixon and Markezich (1992) the only key characters to distinguish *Erythrolamprus poecilogyrus poecilogyrus* from *Erythrolamprus poecilogyrus schotti* that do not change with ontogeny are: (1) dorsal transverse bands from head to posterior including the mid-body section in *Erythrolamprus poecilogyrus poecilogyrus* versus changing to a dorsal line or interruption of transverse bands in *Erythrolamprus poecilogyrus schotti*; and (2) dark to black edged cephalic shields in *Erythrolamprus poecilogyrus poecilogyrus* versus relatively featureless cephalic shields in *Erythrolamprus poecilogyrus schotti*. In addition, young (SVL< 350 mm) *Erythrolamprus poecilogyrus schotti* from northeastern Brazil are characterized by widely spaced mid-dorsal marking.

In our study area, offspring and juvenile specimens were found displaying characters of color pattern of both taxa noted above, whereas other juveniles exhibited widely spaced dorsal markings and some specimens showed intermediate expression of dark-edging of cephalic shields. This pattern concurs with the polymorphic scenario evaluated by Fernandes (2006), where the pattern of young *Erythrolamprus poecilogyrus schotti* predominates in northeastern Brazil, but with some juveniles exhibiting characters of *Erythrolamprus poecilogyrus poecilogyrus* (from 16% with conspicuous transverse dorsal bands to 74% with well-defined ventral markings). In contrast stands the color pattern variation of adults, whereby *Erythrolamprus poecilogyrus schotti* and *Erythrolamprus poecilogyrus poecilogyrus* separate well at or near the Jequitinhonha River, ca. 16° latitude at Belmonte, Bahia (Fernandes 2006). From there north, only *Erythrolamprus poecilogyrus schotti* adult color pattern occurs including our study region north of Salvador. This demarcation between these two taxa is consistent with a decrease in the number of ventral scales and maxillary teeth from *Erythrolamprus poecilogyrus poecilogyrus* (south) to *Erythrolamprus poecilogyrus schotti* (north) found by Dixon and and Markezich (1992). Hence, we conclude that the *Erythrolamprus poecilogyrus poecilogyrus*-like color pattern in some offspring and juveniles from north of Salvador has been and still is part of their regional morphological variation, and is not related to true *Erythrolamprus poecilogyrus poecilogyrus*, which does not occur in our study area. A first hypothesis states, that the *Erythrolamprus poecilogyrus poecilogyrus*-like juvenile color pattern represents simply an ancestral trait, still occurring in small proportions (i.e. has not been lost through selection or genetic drift) in populations in northeastern Brazilian populations of *Erythrolamprus poecilogyrus schotti*.

A second hypothesis includes a historical component, whereby the occasional occurrence of *Erythrolamprus poecilogyrus poecilogyrus* color pattern characters in the *Erythrolamprus poecilogyrus schotti* range reflect a late Pleistocene/Holocene scenario of expansion and contraction of both taxa’s range, or a shifting of their contact and overlap zones, triggered by greater paleoclimatic and vegetation shifts (e.g. Ab’Saber 1977, Xavier et al. 2015). Northeastern Brazil exhibits a mosaic of ecoregions and range limits with taxa and their co-adapted gene complexes associated to Atlantic Forest, Cerrado and Caatinga relicts (e.g., Freitas 2014). Similar to a scenario suggested for the pitvipers *Bothrops lutzi* and *Bothrops erythromelas* above, a period with dry climate within the late Pleistocene 0.63 – 0.11 mya, in particular during the Last Glacial Maximum 12000–18000 years ago (e.g. Ab’Saber 1977; Pennington et al. 2000), may have caused the expansion of *Erythrolamprus poecilogyrus schotti* to current coastal stretches of northeastern Brazil as far south as Belmonte, Bahia. The occurrence of mainly *Erythrolamprus poecilogyrus schotti* morphology along the coast of northeastern Brazil indicates, that this species of Cerrado and Caatinga (at least for the northern half of Brazil) has adapted to environmental conditions of the Atlantic Forest. This is possibly facilitated by genetic exchange with *Erythrolamprus poecilogyrus poecilogyrus* (an Atlantic Forest species) during postglacial warming from their Pleistocene refuges of Atlantic Forests along the coast (Carnaval and Moritz 2008) or a northward introgression of *Erythrolamprus poecilogyrus poecilogyrus* from its core area south of Belmonte. The few color pattern characters by *Erythrolamprus poecilogyrus poecilogyrus* in the *Erythrolamprus poecilogyrus schotti* range would be such imprints of historical introgression.

As a consequence, the area around Salvador exhibits a large polymorphism in *Erythrolamprus poecilogyrus*, with specimens of each parental form, and those with prominent or only a few intermediate characters, or a combination of distinctive characters from each species, similar as was described in hybrid zones and the occurrence of relictual introgressed morphological characters in North American watersnakes (Mebert 2008, 2010). As Dixon and Markezich (1992) noted, character gradients between *Erythrolamprus poecilogyrus schotti* and *Erythrolamprus poecilogyrus poecilogyrus* in northeastern Brazil are less steep than in southeastern Brazil, which supports the aforementioned relatively recent, late Pleistocene and postglacial expansion/contraction events between these two taxa. However, the occurrence of *Erythrolamprus poecilogyrus poecilogyrus* color pattern characters in more western dry areas of Minas Gerais, Bahia, and Piauí is rather in support of the first hypothesis in regards to color pattern (*Erythrolamprus poecilogyrus poecilogyrus* is an ancestral trait persisting in *Erythrolamprus poecilogyrus schotti* populations), which may not restrict gene flow between both taxa along the southern Bahia coast.

The third species in need of discussion relates to *Erythrolamprus aesculapii* ssp. Peters and Orejas-Miranda (1970) attribute five subspecies to *Erythrolamprus aesculapii*, with two possibilities for the state of Bahia (Curcio 2008): *Erythrolamprus aesculapii monozoa* and *Erythrolamprus aesculapii venustissimus*. *Erythrolamprus aesculapii monozoa* does not apply to our specimen according to inter-ring distances. Freitas (2014) recorded *Erythrolamprus aesculapii aesculapii* also from Lauro de Freitas, but it is unlikely since it is allocated to the Amazon population of the species. *Erythrolamprus aesculapii aesculapii* differs from *Erythrolamprus aesculapii venustissimus* by a light head color with dark body bands and red rings of same size as black rings, whereas *Erythrolamprus aesculapii venustissimus* presents a blackish head with light body bands and red rings twice the size of black rings (Peters and Orejas-Miranda 1970). Therefore, following coloration and geographic distribution, we considered *Erythrolamprus aesculapii venustissimus* more likely to represent the regional *Erythrolamprus aesculapii*. The taxonomy of this species is confusing and its population’s status should be reviewed soon (F. Curcio, pers. comm.).

When we compare the snake species richness of the northern coast of Bahia with other snake assemblages from studies with similar sampling effort and field days, the region exhibits a higher species richness (see Table 3). Guedes et al. (2014) gathered voucher specimens from the entire Caatinga domain, resulting in 112 species. The Serra do Mar region extends along the coast from the state of Rio de Janeiro to Santa Catarina, containing 74 snake species (Marques et al. 2001). Santos-Costa et al. (2015) reported 53 species from a large area within Amazon forest, while Argôlo (2004) obtained 61 species from the southern coastal region of Bahia, covering several types of habitats.

**Table 3.** Richness of snake species: comparison between the northern coast of Bahia and other regions of Brazil.

References	Richness	Region
Guedes et al. (2014)	112	Caatinga domain
Marques et al. (2001)	74	Serra do Mar (SdM)-RJ-SP-PR-SC
Argôlo (2004)	61	Atlantic forest of South of state of Bahia-BA
Santos-Costa et al. (2015)	52	Floresta Nacional de Caxiuanã-PA
**This study**	**49**	**North coast of Bahia**
França and Braz (2013)	47	Parque Nacional Chapada dos Veadeiros-GO
Loebmann and Haddad (2010)	44	Planalto do Parnaíba-CE
Sawaya et al. (2008)	36	Estação Ecológica de Itirapina-SP
Hartmann et al. (2009a)	27	Núcleo Santa Virgínia, SdM-SP
Marques and Sazima (2004)	25	Estação Ecológica Juréia Martins-SP
Miranda et al. (2012)	24	Parque Nacional dos Lençóis Maranhenses
Hartmann et al. (2009b)	24	Núcleo Picinguaba, SdM-SP
Strussmann and Sazima (1993)	22	Pantanal
Mesquita et al. (2013)	22	Caatinga-CE

Stomach content in eleven species was found with lizards representing 40% of food items, amphibians 20%, mammals and snakes 15% each, fishes and invertebrates 5% each. Some of these natural history notes are complemented by references. For example, Mesquita et al. (2011) observed *Philodryas nattereri* to be more active during the warmest period of the day, as well as the ingestion of *Ameivula ocellifera*, which probably is a common prey since both species are abundant along the coastal region. In contrast, most other species recorded at mid-day were in vegetation or in water, likely due to cooler ambient temperatures. In regards to our night observations, the principal activity of *Helicops angulatus* at the margin of lakes and ponds related to the foraging and predation of *Leptodactylus natalensis*, confirming that this snake occasionally feeds on frogs (Martins and Oliveira 1998). Species belonging to the genus *Chironius* are diurnal predators specialized on frogs, confirmed by us through an unidentified tadpole (total lenght = 39.4 mm) that was ingested by a juvenile *Chironius exoletus* (SVL = 190 mm) (Dixon et al. 1993, Pinto et al. 2008). However, the ingestion of a *Hemidactylus mabouia* by *Chironius flavolineatus* could also represent an opportunistic, likely nocturnal predation event. Alencar et al. (2012, 2013) state that lizards are the most frequent food item of *Oxyrhopus trigeminus*, ﻿but Bernarde and Abe (2010) classified it as a nocturnal generalist. However, our survey and predation records show the species may also forage during day, as we found two diurnal lizard species in its diet, *Ameivula ocellifera* and *Tropidurus hygomi*. Alternatively, these lizards were found sleeping and preyed on during nocturnal foraging. Ophiophagy is also common in the genus *Micrurus* (Campbell and Lamar 2004), yet there was no previous record of *Micrurus ibiboboca* ingesting *Amerotyphlops brongersmianus*. We also report the first cannibalism event of *Oxyrhopus trigeminus* in which a specimen (SVL = 390 mm) ingested a juvenile (SVL approximately 140 mm). The ingestion of rodents by vipers is widely reported (Martins and Oliveira 1998, Argôlo 2004) and is confirmed herein with a rat (*Rattus* sp.) ingested by a juvenile *Crotalus durissus cascavela* (SVL = 275 mm) and two *Rattus norvegicus* ingested by adults of *Bothrops leucurus*. But we also detected the remnants of the lizard *Tropidurus hispidus* in the stomach of *Bothrops leucurus* near the Instituto da Mata. *Sibynomorphus neuwiedi* feeds exclusively on slugs (Maia-Carneiro et al. 2012), which was also confirmed in this study, similar to the ingestion of fish by *Helicops leopardinus* (Ávila et al. 2006), for which we recorded the fish *Geophagus brasiliensis* as prey.

### Aspects of snake conservation on northern coastal Bahia

The herpetofauna of the north coast of Bahia is mainly threatened by habitat loss and degradation, including the ongoing expansion of residential areas and hotel-resorts (Tinôco 2011). IUCN (2014) lists only *Siphlophis compressus*, *Thamnodynastes pallidus*, *Erythrolamprus viridis*, *Bothrops erythromelas*, *Bothrops lutzi* and *Crotalus durissus* as species of Low Concern. The threat of habitat loss is listed for *Siphlophis compressus*, whereas the status of *Erythrolamprus viridis* only relates to Caatinga specimens, and is not including *Erythrolamprus viridis viridis* found in the Atlantic forest.

Another deleterious aspect to snake conservation is the negative human perception towards snakes. Religious beliefs and superstition transcend an ancient fear about these animals, combined with the lack of contemporary knowledge (Greene 1997, Shine and Bonnet 2000, Fernandes-Ferreira et al. 2011), which results in countless killing of snakes including non-venomous and mimetic species. The presence of snakes around human habitation often leads to the death of these animals due to the prejudiced perception and low education of residents (Moura et al. 2010). In this study, we observed animals with damaged heads and bodies, mainly viperids (*Bothrops erythromelas*, *Bothrops leucurus*, *Bothrops lutzi*), but also colubrids and dipsadids (*Chironius flavolineatus*, *Erythrolamprus almadensis*, *Leptodeira annulata*, *Oxyrhopus trigeminus*, *Oxyrhopus petolarius*, *Philodryas olfersii*, *Spilotes pullatus* and *Micrurus ibiboboca*). Furthermore, accidental and intentional road killing are additional factors that affect snake populations negatively (Weatherhead and Madsen 2009).

Twelve species were classified as common or intermediately frequent. Since snakes are difficult to sample (Sawaya et al. 2008, Gaston and Fuller 2007), this emphasizes that common and easily sampled species are relevant to better understand the biology of snakes and to better contribute to the conservation of a region as a whole. This study attempts to report for the first time all known snake species from the northern coast of Bahia. This region exhibits a snake species diversity as high as the southern part of the state of Bahia, which likely is the consequence of its distinct mosaic nature of habitat related species assemblages, including species from three distinct morphoclimatic domains, Cerrado, Caatinga, and Tropical Atlantic domains (Xavier et al. 2015). The 17 undetected species during the three years of fieldwork might have become extinct in the region, or simply require more field effort to be detected. Nonetheless, it reflects the lack of knowledge about the status of the northern coast of Bahia. We recommend continuation of monitoring programs, especially those of the forest fragments and inner portions of the region. This will provide an updated overview on the suggested extinct or threatened species, as well as all other species recorded here for the first time and new records.

### Key to snake species from northern coastal Bahia

**Table:** 

1	Rudimentary eyes; ventral and dorsal scales of equal size	**Typhlopidae (*Amerotyphlops brongersmianus*)**
–	Developed eyes; ventral scales larger than dorsals	**2**
2	Presence of loreal pit; solenoglyph dentition; keeled scales	**Viperidae**
–	Absence of loreal pit	**3**
3	Robust body; small undifferentiated cephalic scales; more than 30 dorsal scales	**Boidae**
–	Distinctive cephalic scales	**4**
4	Proteroglyph dentition	**Elapidae**
–	Aglyph or opisthoglyph dentition	**Colubridae** and **Dipsadidae**

**Viperidae**

**Table:** 

1	Tail tip with distinguished structures	**2**
–	Tail tip undistinguished; postocular stripe present	**3**
2	Tail with rattle; brown arrow shaped dorsal scales with white bordered and dark center diamond shape	***Crotalus durissus cascavela***
–	Tail tip with spiked scales; orange coloration with dark diamond blotches along the body; postocular stripe present	***Lachesis muta rhombeata***
3	Dorsal scales with 19 or 21 rows; Pale brown coloration, reddish blotches half-moon shaped with circular spots on margin; fewer than 158 subcaudals	***Bothrops erythromelas***
–	Dorsal scales with 21 or more rows	4
4	Dorsal coloration varies from grayish to dark brown; Dark dorsal blotches bow shaped bordered by 1–2 white scales and separated by circular blotches; venter cream and pigmented; Immaculate supralabials. 172–212 ventrals	***Bothrops leucurus***
–	Ground color grayish with elongated dark blotches half-moon shaped; Occasional white spots on supralabials; 153–156 ventrals for three specimens from north of Salvador (elsewhere 161–179, Campbell and Lamar 2004)	***Bothrops lutzi***

**Boidae**

**Table:** 

1	Labial pits present; dark stripes on head	**2**
–	Labial pits absent	**3**
2	Deep labial pits; variable coloration, usually brown, with dark round blotches along the body; long and prehensile tail; higher than 105 subcaudals	***Corallus hortulanus***
–	Shallow labial pits; amber coloration with lighter ocelli on dorsum; lateral white and brown stripes present at 1/3 of the body; lateral ocelli uniform of brownish coloration and white spots on top; 40–60 subcaudals	***Epicrates assisi***
3	Large sized snake; olive green dorsal coloration with black ocelli along the body; venter yellowish and pigmented	***Eunectes murinus***
–	Grayish coloration with brown blotches; lateral brown ocelli; tail with reddish brown blotches separated by a white stripe; postocular stripe present	***Boa constrictor constrictor***

**Elapidae**

**Table:** 

1	Monadal pattern with white or yellowish edges of black rings separated by red rings; black head cap from rostral to parietals; tail with black and white rings	***Micrurus corallinus***
–	Triad pattern separated by red rings; black rings of similar size intercalated with white rings; 8–12 triads on body; divided cloacal plate; 212–244 ventrals; 18–30 subcaudals	***Micrurus ibiboboca***

**Colubridae and Dipsadidae**

**Table:** 

1	Even dorsal scale rows	**2**
–	Odd dorsal scale rows	**6**
2	Higher than 14 scale rows; apical pits presents; round pupil; black coloration and yellowish venter occasionally reaching dorso-lateral region	***Spilotes pullatus pullatus***
–	Fewer than 12 scale rows; keeled paravertebral scale rows; light vertebral stripe present	**3**
3	Brown head; grayish-black coloration bordering the light stripe up to 1/3 of body; brown on the remaining body; 12/12/8 scale rows, rarely 12/12/10	***Chironius flavolineatus***
–	Coloration not as above	**4**
4	12/12/10 scale rows; olive dorsum; light stripe bordered by black line; yellowish venter from labials; divided subcaudals with dark stripe between them	***Chironius bicarinatus***
–	12/12/8 or 14/12/8 scale rows	**5**
5	Olive dorsum at 1/3 of the body and brownish on the remaining portion; paravertebrals in lighter coloration; yellowish venter up to labials; dark stripe dividing dorsals and subcaudals; 132–144 ventrals	***Chironius exoletus***
–	Dark olive dorsum, lighter towards the venter; white chin; yellow venter; dorsal scales on tail with yellow center; 12/12/8 or 14/12/8 scale rows	***Chironius carinatus***
6	17 or less scale rows at midbody	**7**
–	19 or more scale rows at midbody	**20**
7	15 scale rows on midbody	**8**
–	17 scale rows on midbody	**14**
8	15/15/11 scale rows; keeled dorsal scales; metallic green with brown vertebral stripe; dark stripe above supralabials up to the end of head; big eye with round pupil	***Leptophis ahaetulla liocercus***
–	15/15/15 scale rows	**9**
9	Two black ring pattern separated by red rings; black rings separated by white or yellow rings; big and dark eyes; opisthoglyph dentition; 179–195 ventrals; 35–46 subcaudals	***Erythrolamprus aesculapii venustissimus***
–	Brown or green coloration, never red	**10**
10	Brownish head and dorsum; eliptical pupil; dark brown bands on dorsum; 163–182 ventrals; 59–77 subcaudals; single anal plate	***Sibynomorphus neuwiedi***
–	Ocelli or uniform pattern; divided anal plate	**11**
11	Brown head; Ocelli behind the head up to midbody; dorsum greyish to reddish-brown; yellowish venter; 160–173 ventrals; 70–77 subcaudals	***Taeniophallus occipitalis***
–	Not as above	**12**
12	Body uniform brown; pigmented head and nucal stripe; vertebral stripe; small eye; 138–156 ventrals; 50–63 subcaudals	***Tantilla melanocephala***
–	Uniform, ocelli or dark bands pattern	**13**
13	Dark brown or dark green coloration; juveniles present brown or redish bands separated by white or cream stripes; 157–180 ventrals; 86–110 subcaudals	***Drymoluber dichrous***
–	Brown head; dorsum light brown with dark ocelli; lateral blotches of the same color; venter lightly pigmented; ≥ 165 ventrals; ≥ 87 subcaudals	***Mastigodryas bifossatus***
14.	Without scale row reduction; vertebral scales modified; slender body; head strongly distinct; big eyes and elliptical pupil; brownish coloration with dark diamond shape blotches	***Imantodes cenchoa cenchoa***
–	With scale row reduction	**15**
15	17/17/13 scale rows	**16**
–	17/17/15 scale rows	**17**
16	Elongated head and slender body; big eyes with round pupil; brown coloration; chin and ventral gular portion yellowish or white; Dark oral mucosa	***Oxybelis aeneus***
–	Smooth scales; single anal plate; 2+2/2+3 temporals; postocular brown stripe; yellow gold coloration with black and yellow spots up to midbody	***Thamnodynastes pallidus***
17	Single anal plate; juveniles present yellowish coloration with darker transversal bands; adults present approximately dark anterior up to 2/3 of the body and yellow to the remaining portion; yellow venter	***Drymarchon corais corais***
–	Divided anal plate	**18**
18	Green-brownish coloration; single apical pit present; dorsolateral stripes from midbody to the end of tail; supralabials, chin and venter yellowish; Venter with alternating dark bands	***Erythrolamprus reginae semilineatus***
–	Without apical pits	**19**
19	Head light-brown; supralabials cream with black border; olive dorsum; black nucal stripe followed by a cream stripe; venter yellow-cream with black blotches	***Erythrolamprus miliaris merremi***
–	Dorsum black with transversal cream stripes; supralabials lighter than dorsum; white chin; reddish venter with 25–35 black bands	***Erythrolamprus taeniogaster***
20	Without apical pits	**21**
–	With apical pits	**26**
21	Single internasal scale	**22**
–	Pair of internasal scales	**23**
22	Dorsum light-brown or cream with dark blotches; scales strongly keeled; cream venter with black bands; 112–124 ventrals; 79–99 subcaudals	***Helicops angulatus***
–	Olive coloration with dark ocelli; scales weakly keeled; red venter with black bands; white chin; 121–133 ventrals; 57–79 subcaudals	***Helicops leopardinus***
23	Brown coloration lighter on the head; white or cream supralabials, chin and venter; modified rostral scale; smooth scales	***Phimophis guerini***
–	Regular rostral scale	**24**
24	Green coloration; juveniles present dark green with black spots along the body; supralabials, chin and anterior portion of body yellowish; 19/19/17 scale rows; >176 ventrals	***Erythrolamprus viridis viridis***
–	Other color pattern	**25**
25	Top of the head brown with a cream or white V or Y shape mark; Dorsum present brownish bands divided by grayish stripes; dorsolateral cream stripes; orange-reddish venter with black bands; 19/19/17 scale rows	***Erythrolamprus almadensis***
–	19/19/15 scale rows; Brownish coloration, occasionally with diagonal black ribs; cream venter weakly pigmented	***Erythrolamprus poecilogyrus schotti***
26	Single apical pit	**27**
–	Two apical pits	**30**
27	Body uniform green, venter in lighter coloration; long tail; divided anal plate; 164–208 ventrals; 92–132 subcaudals	***Philodryas olfersii herbeus***
–	Brown pattern, never green	**28**
28	Brown head; coloration varies from uniform brown to grayish; White venter; divided anal plate; 166–189 ventrals; 76–113 subcaudals	***Philodryas patagoniensis***
–	19/19/17 scale rows arranged in oblique rows	**29**
29	Head with small blotches and stripes from internasals to postocular portion; brown coloration with bow shape blotches connected bilaterally or usually alternated	***Xenodon merremii***
–	Single blotch on head bifurcated on the neck; Brown dorsum with dark bow shape blotches along the body; pigmented venter	***Xenodon rhabdocephalus rhabdocephalus***
30	Modified vertebral row; orange head; juveniles present white stripe on parietals; black nucal stripe and red-wine coloration with black bands; white venter; >230 ventrals; >105 subcaudals	***Siphlophis compressus***
–	Vertebral scales not modified	**31**
31	Keeled scales in 19 or 21 rows; brown head; Internasals, prefrontals and labials pigmented; black postocular stripe present; dark-brown dorsum, yellowish towards the venter with black diagonal stripes	***Spilotes sulphureus sulphureus***
–	Smooth scales	**32**
32	19 or 21 scale rows; brown head, darker on parietals; juveniles present white stripe on parietals; brown coloration with dark blotches	***Leptodeira annulata annulata***
–	Pattern black, brown or with red, black and white rings	**33**
33	21 scale rows; brown head with white stripes above the eyes up to parietals; white supralabials weakly pigmented; brown-grayish coloration with dorsolateral dark stripes and white paraventral stripe; white venter with dark stripe on edge of ventrals	***Philodryas nattereri***
–	19 scale rows, single anal plate	**34**
34	Uniform coloration with or without irregular spots	**35**
–	Pattern of black, red and white rings, white venter	**36**
35	Black or dark-brown coloration; juveniles present a white stripe on head, dark nucal stripe and red-wine coloration; 207–235 ventrals; 53–81 paired subcaudals	***Clelia plumbea***
–	Black coloration with or without white blotches; juveniles present a white stripe on head, dark nucal stripe and red-wine coloration; 188–202 ventrals; 81–97 single subcaudals	***Pseudoboa nigra***
36	Black head up to parietals; supralabials grayish; red nucal stripe; triad of black and white rings separated by red rings; white chin	***Oxyrhopus trigeminus***
–	Black head with red nucal stripe; juveniles present black and white coloration; adults present black and red coloration	***Oxyrhopus petolarius digitalis***

## Appendix 1

Museums examined voucher specimens from the municipalities of the study in alphabetical order.

***Amerotyphlops brongersmianus***: Camaçari: MZUFBA 1725. Conde: CHECOA 2896. Mata de São João: CHECOA 1093, 1304, 1592, 2953, MZUEFS 1421. ***Boa constrictor constrictor***: Camaçari: CHECOA 2777. Catu: CHECOA292. Mata de São João: CHECOA 2778, MZUFBA 1968. ***Bothrops erythromelas***: Camaçari: MZUFBA 499. Lauro de Freitas: MZUFBA 1366. ***Bothrops leucurus***: Camaçari: CHECOA 2875, MZUEFS 381, 882, 1075, MZUFBA 320, 494, 499, 501, 695, 696, 697, 698, 699, 744, 745, 749, 869, 874, 875, 878, 917, 922, 932, 937,939, 940, 996, 1220, 1223, 1225, 1226, 1228, 1239, 1241, 1242, 1249, 1257, 1281, 1901. Catu: MZUFBA 473, 476, 941, 1950, 2411, 2437. Conde: MZUFBA 1255. Dias D’Ávila: MZUEFS 1179, 1180, 1467, MZUFBA 1528, 2409. Entre Rios: MZUEFS 1745. Esplanada: CHECOA 194. Itanagra: MZUFBA 746, 876, 925, 928, 938, 1246. Jandaíra: MZUEFS 8634, MZUFBA 901. Lauro de Freitas: MZUFBA 739, 923, 930, 936, 1250, 1254, 1447, 1633, 1634, 1878, 2020, 2116, 2139, 2151, 2227. Mata de São João: CHECOA 1055, 1251, 1257, 1258, 1302, 1303, 1334, 1335, 1336, 1337, 1338, 1339, 1343, 1345, 1599, 1894, 2468, 2781, 2782, 2783, 2784, 3088, MZUEFS 488, 1021, 1107, 1425, 1426, 1427, 1429, 1507, MZUFBA 420, 495, 693, 752, 812, 931, 933, 1227, 1229, 1240, 1243, 1406, 1526, 1532, 1547, 1885, 1886. Pojuca: MZUFBA 701. Simões Filho: MZUFBA 314, 487, 491, 497, 700, 747, 748, 814, 868, 909, 916, 919, 924, 1001, 1248, 1546, 1876, 1880, 1997, 2112, 2115, 2223, 2438. ***Bothrops lutzi***: Camaçari: MZUFBA 985, 986, 1191. ***Chironius exoletus***: Catu: CHECOA 278, 290, 291, MZUFBA 466, 467. Entre Rios:1591. Mata de São João: CHECOA 1314, 1325, 1327, 1340, MZUFBA 418, 2209. Simões Filho: MZUFBA 619. ***Chironius flavolineatus***: Camaçari: CHECOA 2918, 2998, MZUFBA 1199. Catu: MZUFBA 610. Conde: CHECOA 1589. Dias D’Ávila: MZUEFS 1469. Entre Rios: CHECOA 1583. Itanagra: MZUFBA 401. Lauro de Freitas: MZUFBA 1277. Mata de São João: CHECOA 1297, 1298, 1326, 1328, 1575, 1896, 1897. Simões Filho: MZUFBA 1603. ***Clelia plumbea***: Catu: CHECOA 265, 269, 294. ***Corallus hortulanus***: Lauro de Freitas: CHECOA 2801. ***Crotalus durissus cascavella***: Camaçari: MZUFBA 1003, 1549, 2363. Catu: CHECOA 196, MZUFBA 455. Entre Rios: CHECOA 2859. Esplanada: MZUFBA 907. Lauro de Freitas: MZUFBA 2143, 2148. Mata de São João: CHECOA 1111, 1112, 1344, 2788, 2789. ***Drymarchon corais corais***: Catu: CHECOA 284. Dias D’Ávila: MZUEFS 1466. ***Drymoluber dichrous***: Catu: CHECOA 176, MZUFBA 2156, 2158, 2162. ***Epicrates assisi***: Mata de São João: CHECOA 1613. ***Erythrolamprus aesculapii venustissimus***: Lauro de Freitas: MZUEFS 733. ***Erythrolamprus almadensis***: Camaçari: MZUFBA 833 1284. Catu: CHECOA 276. Dias D’Ávila: MZUEFS 1463, 1464. Lauro de Freitas: MZUEFS 741, 744, MZUFBA 1283. Mata de São João: CHECOA 1418, 1419, MZUEFS: 1423, 1422. Pojuca: CHECOA 239, 240, 241, 242, 243. ***Erythrolamprus miliaris merremi***: Dias D’Ávila: MZUEFS 1172. Pojuca: CHECOA 244. Simês Filho: CHECOA 2912. ***Erythrolamprus poecilogyrus schotti***: Camaçari: MZUFBA 300. Catu: CHECOA 251, 268, MZUFBA 478, 765, 538. Mata de São João: CHECOA 1577, MZUFBA 410, 423, 424, 425, 426. Lauro de Freitas: MZUEFS 739. Pojuca: CHECOA 222, 223, 224, 228, 229, 230, 245, 246. ***Erythrolamprus reginae semilineatus***: Catu: CHECOA 293, MZUFBA 487,288. Mata de São João: CHECOA 2588. Pojuca: CHECOA 234, 235, 236, 237. ***Erythrolamprus taeniogaster***: Camaçari: MZUFBA 563. Catu: CHECOA 270, 271, 272, MZUFBA 474, 479. Lauro de Freitas: MZUFBA 1559, 2484. Mata de São João: CHECOA 1593, 2791, MZUFBA 1640. Simões Filho: MZUFBA 608. ***Erythrolamprus viridis viridis***: Mata de São João: MZUFBA 429. ***Eunectes murinus***: Camaçari: MZEFS 1074. Mata de São João: MZUFBA 2395. ***Helicops angulatus***: Camaçari: CHECOA 2904, MZUFBA 333. Catu: CHECOA 255, 256, MZUFBA 461, 163, 2159. Mata de São João: CHECOA 1895, 2773. Simões Filho: MZUFBA 1935. ***Helicops leopardinus***: Camaçari: MZUFBA 1939. Catu: CHECOA 279, 280, MZUFBA 462. Lauro de Freitas: MZUEFS 1044, MZUFBA 1286, 2140, 2152. Mata de São João: CHECOA 1247, 1260. Pojuca: CHECOA: 225, 231, 232. ***Imantodes cenchoa cenchoa***: Mata de São João: CHECOA 2883. Simões filho: MZUFBA 2867. ***Leptophis ahaetulla liocercus***: Catu: MZUFBA 2219. Mata de São João: CHECOA 2952. Simões Filho: MZUFBA 510. ***Leptodeira annulata annulata***: Camaçari: MZUEFS 1090, MZUFBA 633. Catu: MZUFBA 570. Entre Rios: CHECOA 1585, MZUEFS 1744, 1746. Jandaíra: CHECOA 2965. Lauro de Freitas: MZUEFS 693, MZUFBA 493. Mata de São João: CHECOA 1089, 1255, 1330, 1331, 1332, 1341, 1590, 1601, 2779, 2780, MZUEFS 1428. ***Mastigodryas bifossatus***: Catu: MZUFBA 841. Jandaíra: MZUESC 3238. Lauro de Freitas: MZUFBA 569. Mata de São João: MZUFBA 422. ***Micrurus ibiboboca***: Camaçari: CHECOA 743, 2909, MZUFBA 403, 773, 787, 1016, 1276, 1358, 1388, 1635, 2334, 2335. Catu: CHECOA 193, 207, 208, 209, 210, 211, 212, 213, 214, 215, 216, 217, 218, 220, MZUFBA 469, 470, 471, 566, 567, 612, 613, 614, 615, 632. Dias D’Ávila: MZUEFS 1460, 1461, 1462, MZUFBA 371. Entre Rios: CHECOA 1470. Esplanada: CHECOA 2900. Lauro de Freitas: MZUEFS 748, MZUFBA 1441, 1870, 1947, 1948, 1974, 2297, 2404. Mata de São João: CHECOA 1051, 1054, 1077, 1114, 1148, 1293, 1294, 1295, 1410, 1597, 1602, 1604, 1606, 1607, 1608, 2115, 2792, 2966, 3091, 3113, MZUFBA 409, 436, 450, 980, 1462, 1972, 2237, 2454. Simões Filho: MZUEFS 516, 1050, MZUFBA 636, 772, 774, 813, 2373. ***Micrurus corallinus***: Simões Filho: MZUFBA 997. ***Oxybelis aeneus***: Catu: CHECOA 286, MZUFBA 472, 2157. Mata de São João: CHECOA 1048, 1254. ***Oxyrhopus petolarius digitalis***: Camaçari: MZUFBA 1285, Catu: MZUFBA 764. Mata de São João: CHECOA 88, 2964, 3092. ***Oxyrhopus trigeminus***: Camaçari: CHECOA 2910, 2911, MZUFBA 375, 384, 444, 629, 1321, 1298. Catu: MZUFBA 468, 475, 2155. Dias D’Ávila: MZUEFS 1465. Entre Rios: CHECOA 1584. Lauro de Freitas: MZUEFS 745, MZUFBA 343, 417, 786, 943, 1932, 1942. Mata de São João: CHECOA 1146, 1147, 1253, 1296, 1316, 1317, 1318, 1415, 1582, 1594, 1596, 1598, 1600, 2790, MZUEFS 437, 855, MZUFBA 413, 414, 415, 416. Simões Filho: MZUFBA 443. ***Pantherophis guttatus***: Camaçari: CHECOA 142.

*Phimophis guerini*: Camaçari: MZUFBA 1960, 2471. Mata de São João: CHECOA 1411, 1420. ***Philodryas nattereri***: Camaçari: MZUFBA: 1961. Catu: MZUFBA 465. Mata de São João: CHECOA 1305, 1320, 1321, 1322, 1587, MZUFBA 1431, 2238, 2371. ***Philodryas olfersii herbeus***: Camaçari: CHECOA 2421, 2906. Catu: CHECOA 257, 266. Dias D’Ávila: MZUFBA 1426. Jandaíra: 2527. Lauro de Freitas: MZUFBA 643, 2341, 2491. Mata de São João: CHECOA 1052, 1062, 1299, 1300, 1301, 3089. ***Philodryas patagoniensis***: MZUEFS 1407. Camaçari: CHECOA 1109, 1410, 2884, 2908, MZUFBA 356, 1275, 1282, 1297, 1364, 1491, 1492, 2282. Catu: CHECOA 258, MZUFBA 1281. Lauro de Freitas: MZUFBA 1278, 1294. Mata de São João: MZUEFS 599. ***Pseudoboa nigra***: Camaçari: CHECOA 1053, MZUFBA 492, 1323, Catu: MZUFBA 484, 485. Mata de São João: MZUFBA 421, 2435. ***Sibynomorphus neuwiedi***: Catu: CHECOA 252, 253, 254, 259, 260, 261, 262, 263, 264, 287, 288. Mata de São João: CHECOA 2965, 3090 Pojuca: CHECOA 247. ***Siphlophis compressus***: Mata de São João: CHECOA 2854. ***Spilotes pullatus pullatus***: Camaçari: MZUFBA 2142. Mata de São João: CHECOA 1422, 2785, 2786, MZUEFS 1093, 1307. Simões Filho: MZUFBA 621. ***Taeniophallus occipitalis***: Camaçari: MZUEFS 1308. Mata de São João: CHECOA 1049, 1262, 3093. ***Tantilla melanocephala***:﻿ Camaçari: CHECOA 2065, MZUFBA 1726, 1727. Mata de São João: CHECOA 1256, 1313, 1315, 1342, 1603, 1893, 2798. ***Thamnodynastes pallidus***: Mata de São João: CHECOA 2852. ***Xenodon merremii***: Camaçari: CHECOA 2907, MZUFBA 396, 449, 452, 1311. Catu: CHECOA 283, MZUFBA 456, 458, 459, 460, 464, 477. Dias D’Ávila: MZUEFS 1468. Mata de São João: CHECOA 226, 1306, 2787, MZUFBA 405, 411, 850, 1007, 1437, 1527. Pojuca: MZUFBA 298. ***Xenodon rabdocephalus rabdocephalus***: Camaçari: MZUFBA 442. Catu: MZUFBA 831, 2154.
